# YY1 inhibits differentiation and function of regulatory T cells by blocking Foxp3 expression and activity

**DOI:** 10.1038/ncomms10789

**Published:** 2016-02-19

**Authors:** Soo Seok Hwang, Sung Woong Jang, Min Kyung Kim, Lark Kyun Kim, Bong-Sung Kim, Hyeong Su Kim, Kiwan Kim, Wonyong Lee, Richard A. Flavell, Gap Ryol Lee

**Affiliations:** 1 Department of Life Science, Sogang University, 35 Baekbeom-ro, Seoul 121-742, Korea; 2 Department of Immunobiology, Yale University School of Medicine, New Haven, Connecticut 06520, USA; 3 Howard Hughes Medical Institute, Department of Immunobiology, Yale School of Medicine, New Haven, Connecticut 06520, USA; 4 Department of Rheumatology, Yale University School of Medicine, New Haven, Connecticut 06520, USA; 5 Department of Plastic and Reconstructive Surgery, Hand Surgery—Burn Center, Medical Faculty, RWTH Aachen University, Templergraben 55, 52062 Aachen, Germany; 6 Institute of Biochemistry and Molecular Cell Biology, RWTH Aachen University, Templergraben 55, 52062 Aachen, Germany

## Abstract

Regulatory T (T_reg_) cells are essential for maintenance of immune homeostasis. Foxp3 is the key transcription factor for T_reg_-cell differentiation and function; however, molecular mechanisms for its negative regulation are poorly understood. Here we show that YY1 expression is lower in T_reg_ cells than T_conv_ cells, and its overexpression causes a marked reduction of Foxp3 expression and abrogation of suppressive function of T_reg_ cells. YY1 is increased in T_reg_ cells under inflammatory conditions with concomitant decrease of suppressor activity in dextran sulfate-induced colitis model. YY1 inhibits Smad3/4 binding to and chromatin remodelling of the *Foxp3* locus. In addition, YY1 interrupts Foxp3-dependent target gene expression by physically interacting with Foxp3 and by directly binding to the Foxp3 target genes. Thus, YY1 inhibits differentiation and function of T_reg_ cells by blocking Foxp3.

## Abstract (web summary)

Treg control the magnitude of immune responses, but how these cells are controlled is less understood. Here the authors show that a transcriptional repressor YY1 inhibits Foxp3, the master regulator of Treg, by repressing its transcription, and by directly interacting with Foxp3 and its target gene promoters.

## Graphical abstract

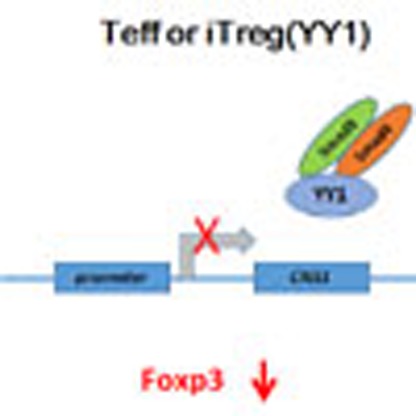

Regulatory T (T_reg_) cells play critical roles in maintaining immune homeostasis. T_reg_ cells inhibit differentiation and proliferation of conventional T (T_conv_) cells including Th1, Th2, Th17 and Tfh cells. T_reg_ cells thereby prevent excessive immune responses against self-antigens, food antigens, commensal microorganisms and cancers^1^,^2^,^3^. T_reg_ cells can develop either in the thymus (tT_reg_) or by differentiation from naïve CD4 T cells in the periphery (pT_reg_).

Foxp3, an X-chromosome-encoded member of the Forkhead family, is the lineage-determining transcription factor for T_reg_ cells^2^,^3^,^4^. Foxp3 is involved in the control of differentiation and function of T_reg_ cells. Loss of Foxp3 function causes the fatal autoimmune disease immune dysregulation, polyendocrinopathy, enteropathy, X-linked in humans and mice^5^,^6^,^7^. Ectopic expression of Foxp3 in CD4^+^CD25^–^ T cells confers suppressive function and induces expression of T_reg_ cell signature genes including *Cd25*, *Ctla4*, *Icos* and *Gitr*^8^,^9^. Furthermore, sustained Foxp3 expression is essential for maintenance of the T_reg_-cell phenotype and associated functions^1^,^10^. Deficiency of *Foxp3* expression in T_reg_ cells causes both defective function of T_reg_ cells and the acquisition of T_conv_-cell properties^5^,^6^,^7^. Taken together, these previous studies show that Foxp3 is indispensable for the differentiation and function of T_reg_ cells, specifying the T_reg_ cell lineage.

Understanding the positive and negative regulation of Foxp3 is critically important in controlling T_reg_ cell-regulated immune responses, including those involved in autoimmune diseases, allergies, organ transplantation and cancer^7^. For example, upregulation of T_reg_ function is likely to be beneficial for autoimmune diseases, allergy and organ transplantation. By contrast, downregulation of T_reg_ function could enhance protective immunity against infectious agents and cancer^7^.

A number of transcription factors play roles in the induction of *Foxp3* and downstream signalling pathways by TCR/CD28 stimulation. For example, at the *Foxp3* locus, NFAT, AP1, SP1 and c-Rel bind to the promoter; AP1 and NFAT bind conserved non-coding sequence 1 (CNS1); CREB and ATF bind to CNS2 and c-Rel binds to CNS3 in response to TCR/CD28 activation^3^,^11^,^12^. interleukin (IL)-2 signalling is important for the induction of *Foxp3* gene by STAT5, which binds to the promoter and CNS2 of the *Foxp3* locus^3^,^11^,^12^. Transforming growth factor (TGF)-β also plays a crucial role in the induction of the *Foxp3* gene. Following TGF-β-induced phosphorylation of Smad3 and its dimerization with Smad4, the heterodimer translocates into the nucleus and binds to CNS1 to induce *Foxp3* gene expression^3^,^4^,^11^,^12^. Other transcription factors including Foxo1, Foxo3, Runx1, Runx3, RXR/RAR and Notch1 were also shown to be involved in the induction of Foxp3 expression^3^,^11^,^13^.

Compared with a large number of positive regulators of Foxp3, only a few negative regulators of Foxp3 are known until now. GATA3, a crucial regulator of Th2 differentiation, binds to the *Foxp3* promoter and represses Foxp3 expression during Th2 differentiation^12^,^14^. In addition, STAT3 competes with STAT5 to bind to the *Foxp3* promoter and CNS2, and represses *Foxp3* expression in response to IL-6 (refs 12, 15). Furthermore, RORγt directly binds to the *Foxp3* promoter and causes loss of *Foxp3* expression during Th17 differentiation^16^.

YY1, encoded by *Yy1*, is a transcription factor that functions either as an activator or repressor depending on the chromatin context^17^,^18^,^19^. YY1 interacts with many factors including transcription factors, co-activators and co-repressors. Previous studies show that YY1 has pleiotropic effects on many different cellular processes, including cell growth and differentiation, apoptosis, development and tumorigenesis^17^,^18^,^19^,^20^. Th2-cell differentiation also involves YY1 (refs 21, 22), which mediates chromatin remodelling and chromosomal looping of the Th2 cytokine locus to regulate Th2 cytokine genes^23^. However, the roles of YY1 in T_reg_-cell function have not been investigated.

In the present study, the role of YY1 on T_reg_-cell differentiation and function was examined. YY1 was selectively downregulated in T_reg_ cells, and YY1 overexpression caused a reduction of Foxp3 expression and a loss of suppressive function during T_reg_ differentiation. YY1 inhibited induction of the *Foxp3* gene by impeding the TGF-β-Smad3/4 signalling pathway. Moreover, YY1 physically interacted with Foxp3 and blocked Foxp3-target genes. These results strongly suggest that YY1 inhibits the differentiation and function of T_reg_ cells by blocking expression of Foxp3 and its target genes.

## Results

### YY1 is expressed at low levels in T_reg_ cells

**Figure 1:**
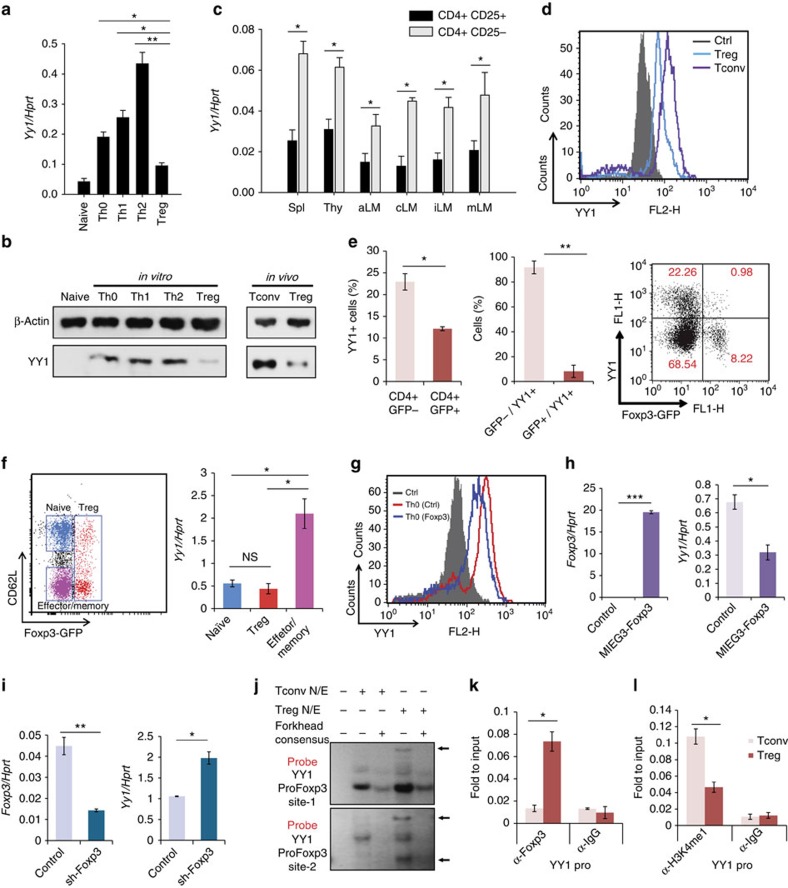
Expression of YY1 is low in T_reg_ cells. (**a**) Naïve CD4 T cells from WT mice were differentiated into Th0, Th1, Th2 and T_reg_ cells for 5 days. Relative amount of *Yy1* transcript was measured by qRT–PCR. (**b**) Relative amounts of YY1 protein in *in vitro*-differentiated CD4 T cells or splenic T_conv_ (CD4^+^CD25^−^) and T_reg_ (CD4^+^CD25^+^) cells were measured by immunoblot analysis. (**c**) Relative amounts of *Yy1* transcript in T_conv_ and T_reg_ cells in axillary (aLN), cervical (cLN), inguinal (iLN) and mesenteric (mLN) lymph nodes and spleen (spl) were detected by qRT–PCR. (**d**) Amounts of YY1 protein in T_conv_ or T_reg_ cells were measured using flow cytometry. IgG: isotype control. (**e**) CD4 cells were enriched from splenocytes of Foxp3-eGFP mice, and then YY1 underwent intracellular staining. The percentage of YY1^+^ cells from CD4^+^GFP^+^(T_reg_) and CD4^+^GFP^−^(non-T_reg_) were shown (left), the percentage of T_reg_ (GFP^+^) and non-T_reg_ (GFP^−^) from YY1^+^ cells were shown (centre) and the FACS plot is shown (right). (**f**) CD4 T cells from Foxp3-eGFP mice were stained with CD62L antibody. Naïve, effector and T_reg_ cells were sorted (left) and relative amounts of *Yy1* transcript were measured by qRT–PCR (right). (**g**) Control GFP vector or Foxp3 expression vector was transduced into Th0 cells. After 4 days, YY1 protein was detected by flow cytometry. IgG: isotype control. (**h**) GFP^+^ cells from **g** were isolated, and relative amounts of *Yy1* and *Foxp3* transcripts were measured using qRT–PCR. (**i**) WT naïve CD4 T cells were transduced with GFP containing control or sh-Foxp3 vectors and differentiated into T_reg_ cells for 4 days. Relative amounts of *Yy1* and *Foxp3* transcripts were measured using qRT–PCR. (**j**) Binding of Foxp3 to two YY1 promoter regions was detected by EMSA using T_conv_ and T_reg_ nuclear extracts with forkhead consensus sequence (x80). Arrows indicate Foxp3-DNA complexes. (**k**–**l**) Binding of Foxp3 (**k**) and H3K4me1 (**l**) in T_reg_ or T_conv_ cells was measured by ChIP assay. Error bars shown in (**a**), (**c**), (**f**), (**h**), (**k**) and (**l**) represent s.d. Statistical differences in (**a**), (**c**), (**f**), (**h**), (**k**) and (**l**) were analysed by Student's *t*-test (*n*=3). *P<0.05, **P<0.01. Experiments were performed at least three times with similar results.

Previous studies identified YY1 as a protein-binding partner^24^ of and the *Yy1* locus as a *cis*-target (binding site) of Foxp3 (refs 24, 25, 26). The role of YY1 in T_reg_-cell differentiation was of particular interest because YY1 expression was selectively low in induced T_reg_ cells compared with *in vitro*-differentiated conventional T (T_conv_) cells (Fig. 1a,b). The expression of YY1 in CD4 T cells isolated from the mouse thymus, spleen and peripheral and mesenteric lymph nodes was then studied. YY1 expression levels were lower in CD4^+^CD25^+^ T_reg_ cells than in CD4^+^CD25^−^ T_conv_ cells from all the lymphoid tissues examined (Fig. 1b–d). The expression levels of YY1 were also investigated in enriched CD4 T cells using Foxp3-eGFP reporter mice (Fig. 1e). The majority of YY1-expressing cells were non-T_reg_ (CD4^+^GFP^−^) cells (Fig. 1e). *Yy1* expression was high in effector/memory CD4 T cells, but low in T_reg_ and naïve CD4 T cells (Fig. 1f).

### Influence of Foxp3 on YY1 expression

To examine whether the low expression of YY1 in T_reg_ cells is caused by Foxp3, overexpression or knockdown (KD) of Foxp3 was used. When murine Th0 cells were transduced with a *Foxp3* overexpression vector (MIEG3-Foxp3) and then cultured for 4 days, levels of YY1 decreased (Fig. 1g,h). Whereas when the cells were transduced with a Foxp3 KD vector (sh-Foxp3) and then cultured for 4 days, the levels of YY1 increased (Fig. 1i). Electrophoresis mobility shift assay (EMSA) and chromatin immunoprecipitation (ChIP) assays using T_reg_ and T_conv_ cells showed that Foxp3 directly bound to the promoter of the *Yy1* gene in T_reg_ cells (Fig. 1j,k), which is consistent with previous Foxp3 ChIP-seq data^24^,^25^,^26^. Two possible Foxp3-binding sites in the promoter are shown in Supplementary Fig. 1. In addition, Histone H3 lysine 4 monomethylation (H3K4me1), a marker for active chromatin, was reduced at the promoter of the *Yy1* gene in T_reg_ cells compared with T_conv_ cells (Fig. 1l). These results suggest that Foxp3 represses the induction of YY1 in T_reg_ cells.

### YY1 inhibits expression of Foxp3 and T_reg_ signature markers

**Figure 2:**
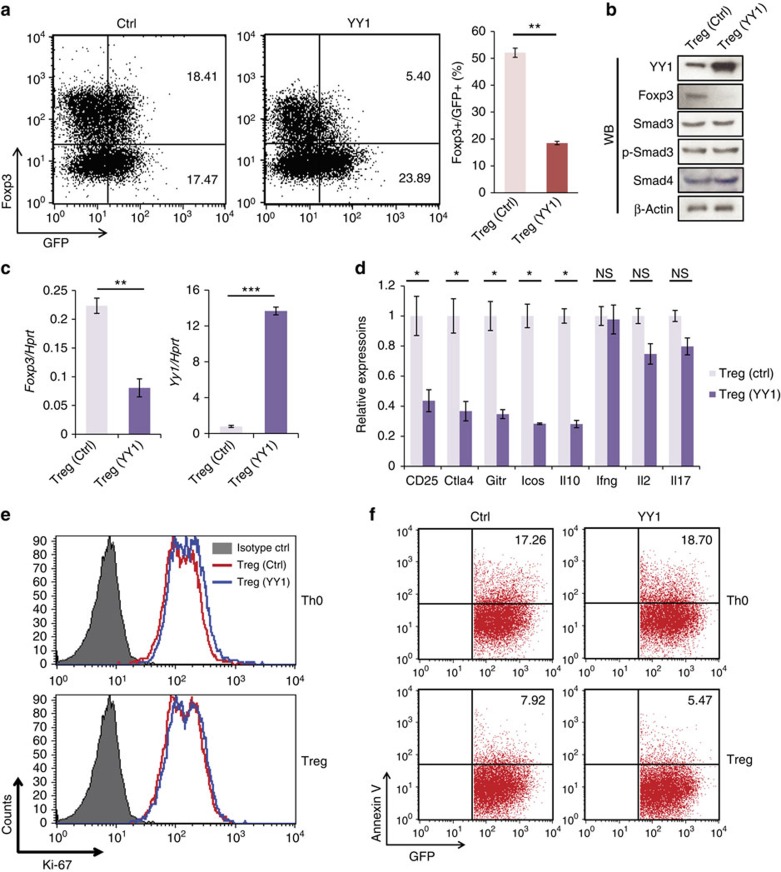
YY1 overexpression causes a loss of Foxp3 and T_reg_ signature genes. (**a**) WT naïve CD4 T cells were transduced with retroviral vector containing *GFP* or *Yy1* and differentiated into T_reg_ cells for 4 days. Expression of Foxp3 was measured by flow cytometry (left), and the ratio of Foxp3^+^ cells in GFP^+^ cells was shown (right). (**b**) Immunoblot analysis of YY1 and Foxp3 from GFP^+^ cells. (**c**,**d**) GFP^+^ cells from **a** were sorted, and total RNA was isolated. Relative amounts of the *Foxp3* and *Yy1* (**c**) and T_reg_ signature genes (**d**) were measured by qRT–PCR. (**e**) Proliferation of control or YY1-transduced Th0 or T_reg_ cells was measured using Ki-67 antibody. Cells were gated on GFP^+^ expression. (**f**) Apoptosis of control or YY1-transduced Th0 or T_reg_ cells was analysed by Annexin V staining. Experiments were performed three times with similar results. Statistical differences in **a** and **d** were analysed by Student's *t*-test (*n*=3). *P<0.05; **P<0.01. Error bars shown in **a** and **d** represent s.d.

**Figure 3:**
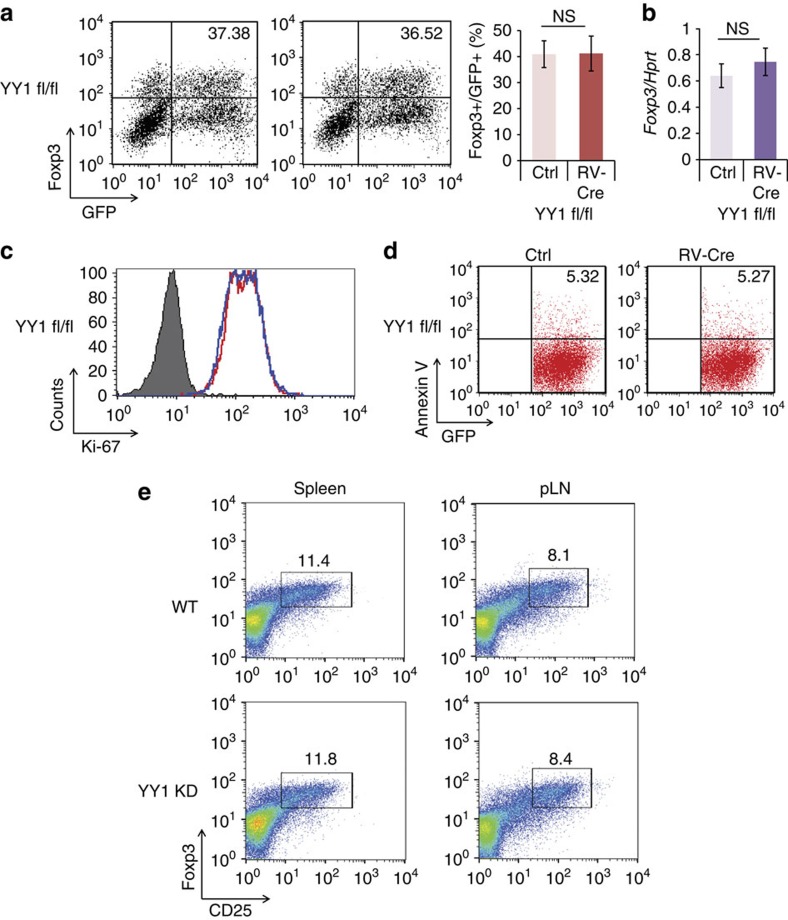
YY1 deficiency does not influence Foxp3 expression. (**a**) Naïve CD4 T cells from YY1 fl/fl mice were transduced with either control vector or CRE-expressing vector (RV-Cre) and cultured under the T_reg_ differentiation condition for 4 days. Foxp3 was stained and measured by flow cytometry. Numbers in the FACS plots indicate the percentage of Foxp3^+^ cells from GFP^+^ cells. (**b**) GFP^+^ cells from **a** were sorted, and total RNA was isolated. A relative amount of *Foxp3* was measured by qRT–PCR. (**c**) Proliferation of YY1-deficient T_reg_ cells was detected with Ki-67 staining from GFP^+^ cells. (**d**) Apoptosis of YY1-deficient T_reg_ cells was measured with Annexin V staining. Experiments were performed five times with similar results. (**e**) T_reg_ populations in the spleen and peripheral lymph nodes of YY1 KD or WT mice were analysed by staining anti-CD4, anti-CD25 and anti-Foxp3. Cells were gated on CD4.

To examine the role of YY1 during T_reg_ differentiation, YY1 was introduced into naïve murine CD4 T cells that were then subjected to T_reg_ differentiation conditions. Foxp3 expression was markedly decreased in YY1-transduced T_reg_ cells compared with those in control vector-transduced T_reg_ cells (Fig. 2a–c). Furthermore, T_reg_ signature genes including *Il10*, *Cd25*, *Ctla4*, *Gitr* and *Icos*, but not unrelated genes including *Ifng*, *Il2* and *Il17*, were also decreased in YY1-transduced T_reg_ cells (Fig. 2d). To get an insight of changes in global gene expression patterns by YY1 expression in T_reg_ cells, a microarray analysis was performed with RNA isolated from cells transduced with control or YY1-expression vector and differentiated into iT_reg_ cells (Supplementary Fig. 2 and Supplementary data; Gene Expression Omnibus (GEO) accession number GSE75052). Overall, among genes changed more than twofold by YY1 overexpression, we found that more genes were upregulated than were downregulated, except genes related to immune responses or inflammatory responses (Supplementary Fig. 2A), suggesting that YY1 enhances general cellular activities. In the category of genes related to T_reg_-cell differentiation, more genes were downregulated than were upregulated by YY1 overexpression (Supplementary Fig. 2B), consistent with our quantitative reverse transcription–PCR (qRT–PCR) data (Fig. 2c,d). Proliferation and cell death of YY1-transduced Th0 and T_reg_ cells were examined using anti-Ki-67 antibody and Annexin V, respectively. Overexpression of YY1 did not affect proliferation or apoptosis in either Th0 or T_reg_ cells (Fig. 2e,f). These data indicate that decreased Foxp3 expression in YY1-transduced T_reg_ cells is not due to proliferation or apoptosis of these cells. To examine whether deficiency influences T_reg_ differentiation, naïve CD4 T cells from floxed YY1 (YY1 fl/fl) mice were transduced with retroviral *Cre*-expression vector (RV-Cre). YY1 deficiency did not influence either the expression of Foxp3 or proliferation and cell death during T_reg_-cell differentiation (Fig. 3a–d). T_reg_ populations in the spleen or peripheral lymph nodes of YY1 KD mice^23^ were not different from those of wild-type (WT) mice (Fig. 3e), suggesting that YY1 KD does not affect Foxp3 expression in T_reg_ cells *in vivo*. Taken together, these data suggest that YY1 inhibits but does not enhance expression of Foxp3 and T_reg_ signature genes in T_reg_ cells.

### YY1 inhibits suppressive functions of T_reg_ cells

**Figure 4:**
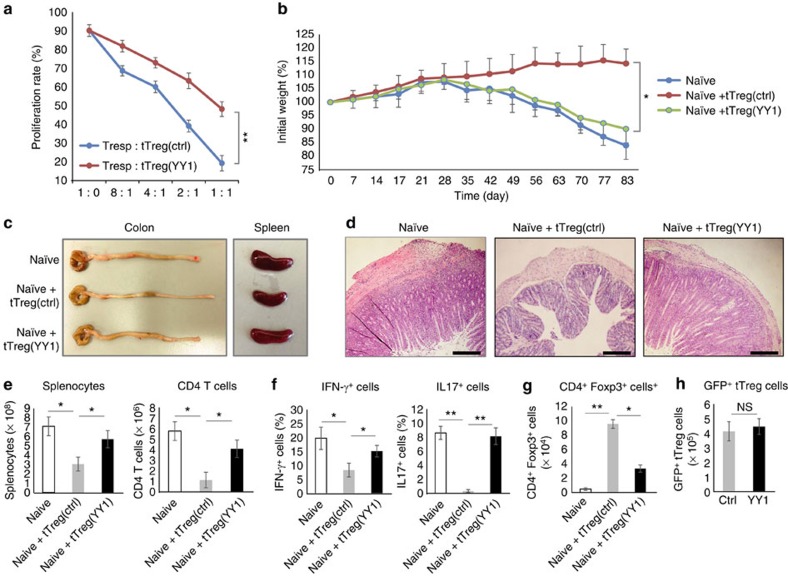
YY1 impairs immunosuppressive function of tT_reg_ cells *in vitro* and *in vivo*. (**a**) *In vitro* immunosuppressive activity of tTreg cells was assessed by proliferation of CD4^+^CD25^−^ Tresp cells (labelled with CFSE from CD45.1^+^ T_resp_ cells). Foxp3^+^ Treg cells were isolated from Foxp3-RFP knock-in mice and transduced with control or YY1-expression vector. GFP^+^ control tTreg (tTreg(ctrl)) or GFP^+^ YY1-overexpressing tTreg (tTreg(YY1)) cells were sorted, mixed with CD4 Tresp cells in various ratios, and cultured in the presence of anti-CD3/CD28 beads for 3 days. Proliferation was measured on CD45.1+ (T_resp_) cells. (**b**–**h**) *In vivo* immunosuppressive activity of tTreg cells was assessed by inflammatory bowel disease model. tT_reg_ (ctrl) and tT_reg_ (YY1) cells were prepared as in **a** from Foxp3-RFP knock-in mice. Naïve CD4 T (CD4^+^CD25^−^CD62L^+^CD45RB^high^) cells alone or together with tT_reg_ (ctrl) or tT_reg_ (YY1) cells were adoptively transferred into RAG1^−/−^ mice. The mice were killed at 12 weeks after the cell transfer, and analysed for disease phenotypes. (**b**) Body weight of the recipient mice was presented as a percentage of the initial weight. (**c**) Gross morphology of colons and spleens. (**d**) Haematoxylin and eosin staining of colon sections. Scale bar indicates 200 μm. (**e**) Absolute numbers of splenocytes and splenic CD4 T cells. (**f**) Frequency of cytokine-producing effector CD4 T cells in the spleen. (**g**) Absolute numbers of CD4^+^Foxp3^+^ cells from the spleen. (**h**) Absolute numbers of GFP^+^ tTreg cells from the spleen. Error bars shown in **b**–**h** represent s.d. Statistical differences in **b**–**h** were analysed by Student's *t*-test (*n*=5). *P<0.05. **P<0.01. NS, not significant.

To examine whether YY1 affects the function of T_reg_ cells, *in vitro* immunosuppression assays were performed. tT_reg_ cells were isolated from Foxp3-RFP knock-in mice^27^ based on red fluorescent protein (RFP) expression and transduced with either control or YY1 expression vector. Murine CD45.1^+^ CD4^+^CD25^−^ responder T (T_resp_) cells stained with carboxyfluorescein succinimidyl ester (CFSE), a fluorescent dye, were mixed with various ratios of control or YY1-overexpressing tT_reg_ cells and incubated for 3 days in the presence of anti-CD3/anti-CD28 beads. Proliferation was measured on CD45.1+ (T_resp_) cells. YY1-overexpressing tT_reg_ cells had weaker suppressive function in proliferation of T_resp_ cells as the ratio increases than control tT_reg_ cells did (Fig. 4a). Next, the role of YY1 in T_reg_ cell function *in vivo* was examined using an animal model of inflammatory bowel disease. CD4^+^CD62L^+^CD45RB^+^ naïve CD4 T cells were adoptively transferred, alone or in combination with control tT_reg_ cells or YY1-overexpressing tT_reg_ cells prepared as described above, into RAG1-deficient mice. As expected, transfer of naïve CD4 T cells alone caused severe colitis characterized by gradual weight loss (Fig. 4b), inflammation in the colonic mucosa, splenomegaly and shortened colon length (Fig. 4c,d). The mice also had an increased total number of splenocytes and CD4 T cells (Fig. 4e), a greater frequency of effector CD4 T cells (Fig. 4f and Supplementary Fig. 3), and a low frequency of Foxp3^+^ cells (Fig. 4g) in the spleen. Transfer of naïve CD4 T cells with control tT_reg_ cells completely suppressed the inflammatory phenotypes; however, the transfer of naïve CD4 T cells with YY1-overexpressing tT_reg_ cells failed to suppress the inflammatory phenotypes (Fig. 4b–g). Adoptively transferred YY1-overexpressing tT_reg_ cells could not inhibit differentiation of the co-transferred naïve CD4 T cells into pathogenic Th1 and Th17 cells, and could not increase their cell number (Fig. 4f,g), suggesting that these tT_reg_ cells are functionally impaired in this experimental setting. The numbers of YY1-transduced tT_reg_ cells recovered in the spleen of the recipient mice were similar to those of control tT_reg_ cells (Fig. 4h), suggesting that the observed phenotypes are not due to poor survival of the YY1-transduced tT_reg_ cells in the mice. Taken together, these data suggest that YY1 inhibits the suppressive function of T_reg_ cells both *in vitro* and *in vivo*.

### YY1 expression is modulated under inflammatory conditions

**Figure 5:**
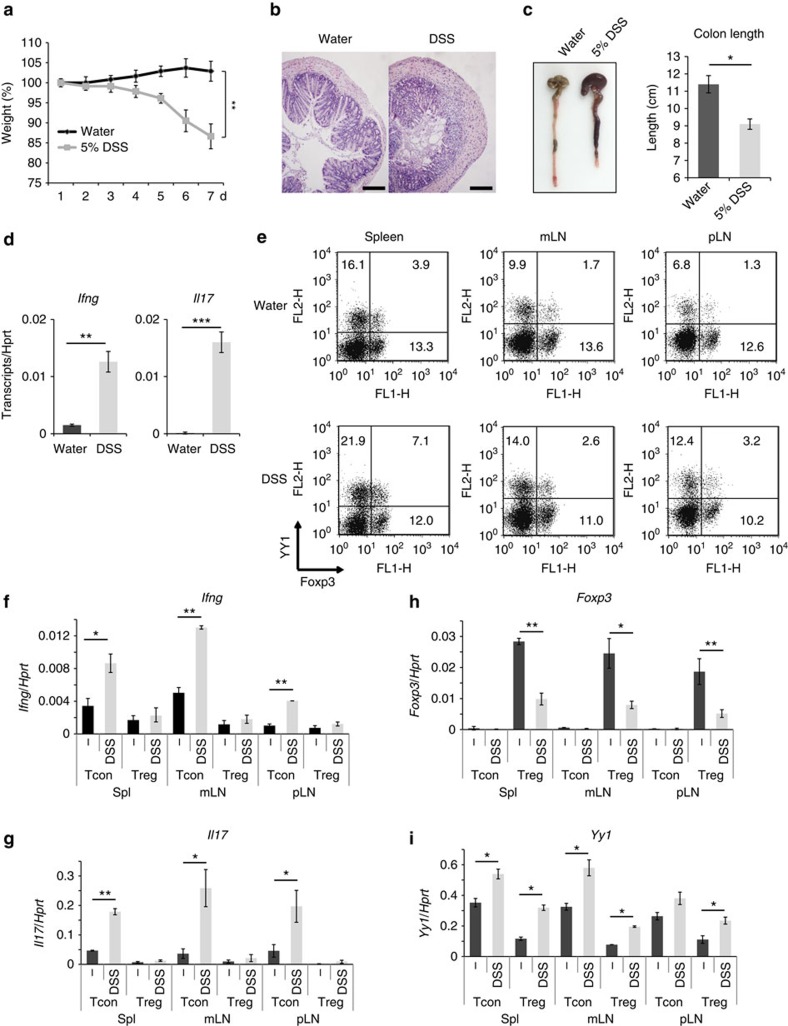
YY1 expression is enhanced in inflammatory condition *in vivo*. WT mice were introduced with water or 5% dextran sodium sulfate (DSS) for 4 days to induce IBD. The mice were killed at 7 days after DSS administration, and analysed for disease phenotypes. (**a**) Body weights of the mice were presented as a percentage of the initial weight. (**b**) Haematoxylin and eosin staining of colon sections. Scale bar indicates 200 μm. (**c**) Gross morphology of colons (left) and colon length (right). (**d**) Colonic RNA was isolated, and relative amounts of *Ifng* and *ll17* transcripts were measured by qRT–PCR. (**e**–**i**) T_conv_ and T_reg_ cells were isolated from the spleen (spl), mesenteric lymph node (mLN) and peripheral lymph node (pLN). Protein levels of YY1 and Foxp3 were measured by intracellular staining (**e**). Total RNA was extracted, and transcripts of *Ifng* (**f**), *Il17* (**g**), *Foxp3* (**h**) and *Yy1* (**i**) were measured by qRT–PCR. Statistical differences in **a**–**i** were analysed by Student's *t*-test (*n*=5). *P<0.05. **P<0.01. ***P<0.001. Error bars shown in **a**–**i** represent s.d.

It has been shown that T_reg_ cell's functional properties can be changed under inflammatory conditions^28^,^29^,^30^. To further investigate physiological relevance of YY1 expression in T_reg_ cells, we examined whether YY1 expression is modulated in T_reg_ cells under an inflammatory condition using dextran sodium sulfate (DSS)-induced colitis model. 5% DSS in drinking water was administered into C57BL/6 mice for 4 days, and the mice were killed at day 7. As was well known, DSS-treated mice had decreased body weights, increased cell infiltration into the colon and shortened colon length (Fig. 5a–c), showing an inflammatory condition in the colon. Cellular RNA was extracted from the colons of the mice and expressions of *Ifng* and *Il17* were measured by qRT–PCR. DSS-treated mice had highly increased *Ifng* and *Il17* (Fig. 5d), showing that inflammatory Th1 and Th17 responses were developed in these mice. Under this condition, the frequencies of YY1-expressing T_reg_ cells were increased in the spleen, mesenteric lymph node and peripheral lymph node in DSS-treated mice compared with those in control mice (Fig. 5e). T_reg_ and T_conv_ cells were isolated from the mice and expression of the *Foxp3*, *Yy1*, *Ifng* and *Il17* transcript was measured by qRT–PCR. Consistent with pathogenic inflammatory condition, expression of *Ifng* and *Il17* was increased in DSS-treated T_conv_ cells (Fig. 5f,g). Expression of *Yy1* was increased in DSS-treated T_reg_ cells concomitant with decreased *Foxp3* expression (Fig. 5h,i). This result suggests that YY1 expression is modulated under inflammatory condition under which T_reg_-cell functions are modulated.

### YY1 inhibits Foxp3 expression by blocking Smad3/Smad4

**Figure 6:**
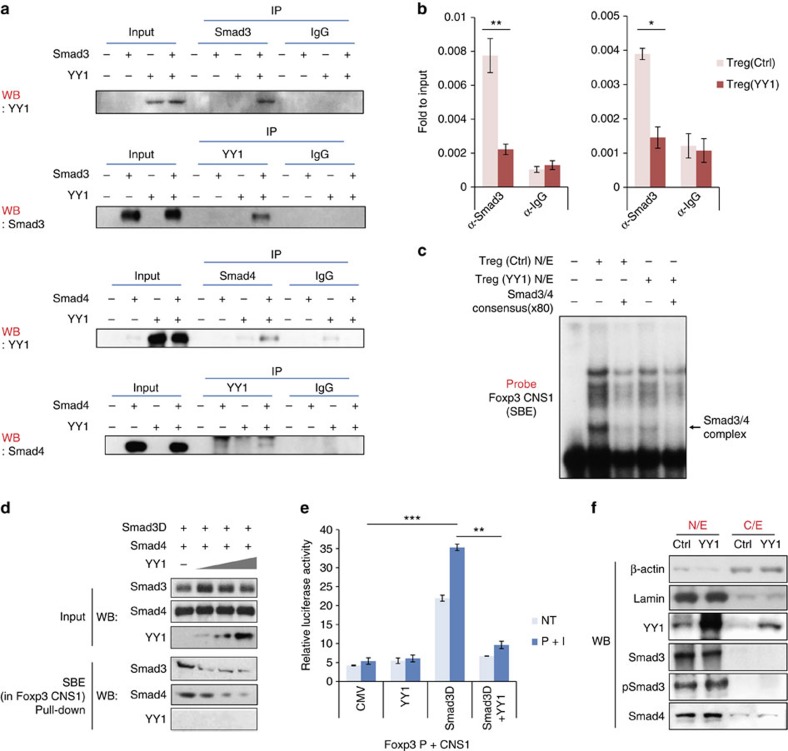
YY1 inhibits expression of Foxp3. (**a**) HEK293T cells were transfected with *Smad3*-, *Smad4*- or *Yy1*-expression vector. Cell lysates were immunoprecipitated with an anti-Smad3, anti-Smad4, anti-YY1 or control IgG antibody. Then, proteins were immunoblotted with an anti-Smad3, anti-Smad4 or anti-YY1 antibody. IP, immunoprecipitation; WB, immunoblot. (**b**) Binding of Smad3 and Smad4 to the Foxp3 CNS1 in control or YY1-expressing T_reg_ cells was measured by ChIP assay. (**c**) Binding of Foxp3 to Smad-binding element (SBE) in the Foxp3 CNS1 was detected by EMSA using control or YY1-expressing T_reg_ nuclear extracts. Competition assay was performed with Smas3/4 consensus sequence (x80). Arrows indicate Smad3/4-SBE complexes. (**d**) Nuclear extracts overexpressing Smad3 and Smad4 with increasing amounts of YY1 were mixed with SBE of the Foxp3 CNS1, and DNA pull-down assay were performed. Oligo-dT was used as a negative control. (**e**) Transactivation activity of the *Foxp3* promoter by Smad3 and YY1 was measured by transient reporter assay. EL4 cells were transfected with Foxp3 promoter-CNS1-luc reporter construct in the absence or presence of *Smad3D-* (constitutively active form of Smad3) and/or *Yy1-*expression vector. I, ionomycin; NT, no treatment; *P*, phorbol 12-myristate 13-acetate. (**f**) Nuclear extract (N/E) and cytosolic extract (C/E) were isolated from control T_reg_ cells or YY1-transduced T_reg_ cells. Relative amounts of YY1, Smad3, pSmad3 and Smad4 in N/E and C/E were measured by immunoblot analysis. β-Actin and Lamin were used as loading controls. Statistical differences in **b**,**d** and **e** were analysed by Student's *t*-test (*n*=3). *P<0.05. **P<0.01. ***P<0.001. Error bars shown in **b**,**d** and **e** represent s.d.

Foxp3 is induced by the TGF-β signalling pathway, and Smad3 and Smad4 are essential transcription factors for the induction of the *Foxp3* gene in this pathway. Upon TGF-β stimulation, the Smad3/4 heterodimer directly binds to the *Foxp3* CNS1, which contains a Smad-binding element (SBE) as a TGF-β sensor^4^. To investigate molecular mechanisms for YY1-mediated Foxp3 regulation, we first examined whether YY1 interacts with Smad3 and Smad4 by co-immunoprecipitation (co-IP) experiments. YY1 was co-immunoprecipitated with Smad3 and Smad4 (Fig. 6a), consistent with a previous study^31^, suggesting that YY1 physically interacts with both Smad3 and Smad4.

To test whether YY1 interferes with the binding of Smad3 or Smad4 to the *Foxp3* CNS1, ChIP assays were performed using a Smad3 or Smad4 antibody in control T_reg_ cells and YY1-transduced T_reg_ cells. Smad3 and Smad4 binding was greatly reduced in YY1-overexpressing T_reg_ cells compared with that in control T_reg_ cells (Fig. 6b). Similar results were obtained with EMSA and DNA pull-down assays, further supporting the possibility that YY1 impedes the binding of Smad3/4 to the SBE in the *Foxp3* CNS1 (Fig. 6c,d).

To examine whether YY1 inhibits transactivation of the *Foxp3* genes by Smad3, transient reporter assays were performed. EL4 cells were transfected with a reporter construct containing Foxp3 promoter linked with CNS1 along with expression vectors of YY1 or Smad3D (constitutively active form of Smad3). As shown previously^4^, Smad3D transactivated the *Foxp3* gene because of the TGF-β sensor located in CNS1. Interestingly, YY1 completely reduced the transactivation of Foxp3 gene even in the presence of Smad3D (Fig. 6e). However, YY1 did not affect heterodimerization of Smad3/4 (Supplementary Fig. 4), expression of Smad3, phospho-Smad3 and Smad4 (Fig. 2c), and nuclear translocation of Smad3/4 complexes (Fig. 6f). Taken together, these data strongly suggest that YY1 inhibits *Foxp3* expression by blocking binding of Smad3/4 to the SBE in the Foxp3 CNS1.

### YY1 binds to and represses the *Foxp3* locus

**Figure 7:**
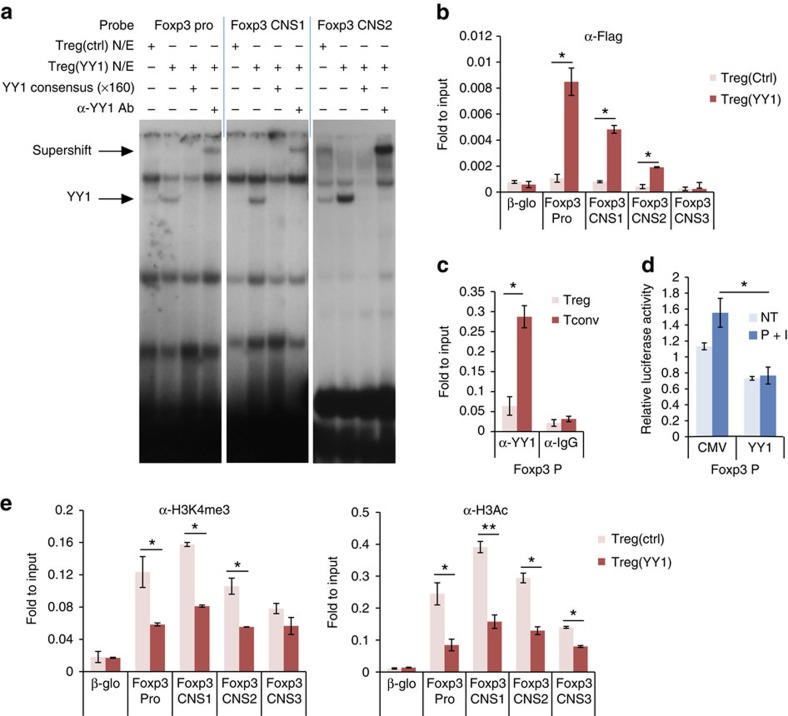
YY1 represses chromatin modification in the *Foxp3* locus. (**a**) Binding of YY1 to YY1-binding sites (YBS) in the Foxp3 promoter (YBS2), CNS1 (YBS4), CNS2 (YBS7) was measured by EMSA using control or YY1-expressing T_reg_ nuclear extracts. Competition assay was performed with YY1 consensus sequence (x160), and supershift assay was performed with an anti-YY1 antibody. (**b**) Binding of YY1-Flag to the Foxp3 locus in control or YY1-Flag-expressing T_reg_ cells was measured by ChIP assay. β-Globin region was used as a negative-binding site. (**c**) Binding of YY1 to the Foxp3 promoter in T_reg_ or T_conv_ cells was analysed by ChIP assay. (**d**) Transactivation activity of the *Foxp3* promoter by YY1 was detected by transient reporter assay. EL4 cells were transfected with Foxp3 promoter-luc reporter construct and YY1-expression vector. I, ionomycin; NT, no treatment; P, phorbol 12-myristate 13-acetate. (**e**) Binding of H3-K4-trimethylation or H3 acetylation to the Foxp3 locus was measured by ChIP assay. β-Globin region was used as a negative-binding site. Values of isotype-matched control IgG were subtracted. Statistical significance of differences from **b**–**e** was determined by Student's *t*-test (*n*=3). *P<0.05. **P<0.01. Data are representative of three independent experiments with similar results. Error bars shown in **b**–**e** represent s.d.

The possibility that YY1 may directly bind to the *Foxp3* locus to repress its expression was tested. Several putative YY1-binding sites were identified within the *Foxp3* locus by transcription factor-binding site analysis (Supplementary Fig. 1). EMSA and ChIP assay in YY1-overexpressing T_reg_ cells confirmed that YY1 directly bound to several regulatory elements at the *Foxp3* locus, including the promoter, CNS1 and CNS2 (Fig. 7a,b). YY1 directly bound to the promoter of the *Foxp3* gene in T_conv_ cells but not in T_reg_ cells (Fig. 7c). Transient reporter assays showed that YY1 inhibited transactivation of the *Foxp3* gene through binding to the *Foxp3* promoter (Fig. 7d).

Chromatin status was investigated in the *Foxp3* locus in control or YY1-overexpressing T_reg_ cells using ChIP assays with anti-H3 acetylation and H3-K4-me3 antibodies. YY1 caused a repressed chromatin status at the *Foxp3* locus (Fig. 7e). Taken together, these data suggest that, when overexpressed, YY1 binds physically to the *Foxp3* locus, inhibits Foxp3 expression and causes a repressive chromatin status at the locus.

### YY1 directly inhibits the expression of Foxp3-target genes

**Figure 8:**
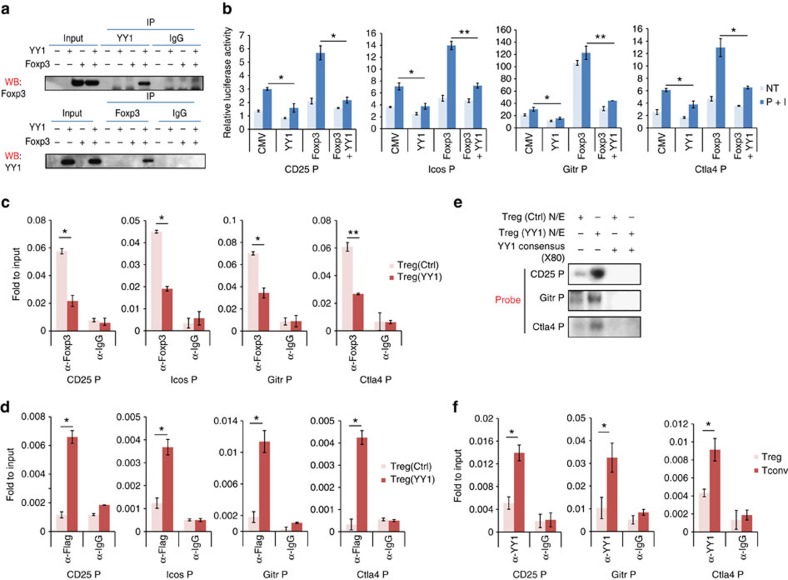
YY1 inhibits expression of Foxp3-target genes. (**a**) HEK293T cells were transfected with a *Foxp3-* or *YY1-*expression vector. Cell lysates were immunoprecipitated with an anti-Foxp3, anti-YY1 or control IgG antibody. Then, proteins were immunoblotted by an anti-Foxp3 or anti-YY1 antibody, as indicated. IP, immunoprecipitation; WB, immunoblot. (**b**) Transactivation activity of YY1 and Foxp3 on the Foxp3-target genes was measured by transient reporter assay. EL4 cells were transfected with CD25P-, IcosP-, GitrP- or Ctla4P-luciferase reporter construct in combination with *Foxp3* or *YY1* expression vector. (**c**,**d**) Binding of Foxp3 (**c**) and YY1-Flag (**d**) to the promoters of the Foxp3-target genes in control or YY1-Flag-expressing T_reg_ cells was measured by ChIP assay. (**e**) Binding of YY1 to YY1-binding sites (YBS) in the promoters of the *CD25*, *Gitr* and *Clta4* genes was detected by EMSA using control or YY1-expressing T_reg_ nuclear extracts. Competition assay was performed with YY1 consensus sequence (x80). (**f**) Binding of YY1 to the promoters of the Foxp3-target genes in T_conv_ or T_reg_ cells was measured by ChIP assay. Error bars shown in **b**–**e** represent s.d. Statistical significance of differences of **b**–**e**) were determined by Student's *t*-test (*n*=3). *P<0.05. **P<0.01. Data are representative of three independent experiments with similar results.

Foxp3 directly binds to and regulates many T_reg_ signature genes, including *Cd25*, *Icos*, *Ctla4*, *Gitr* and *Il10* (refs 25, 26, 32, 33, 34). To test whether YY1 affects this process, the interaction between YY1 and Foxp3 was first examined. Co-IP experiments showed that these proteins interacted with each other (Fig. 8a). Transient reporter assays were then used to examine whether YY1 inhibits transactivation of Foxp3-target genes. EL4 cells were transfected with reporter constructs containing a promoter of *Cd25*, *Icos*, *Gitr* or *Ctla4* together with Foxp3- or YY1-expressing vectors. Foxp3 directly transactivated promoter activities of its target genes (Fig. 8b). However, YY1 expression inhibited this transactivation by Foxp3 (Fig. 8b). Foxp3binding to its target promoters was investigated using ChIP assays in control or YY1-transduced T_reg_ cells. Foxp3 binding to the promoters was remarkably reduced in YY1-transduced T_reg_ cells compared with control cells (Fig. 8c), suggesting that YY1 inhibits Foxp3 binding to its target sites. YY1 directly bound to the promoters of Foxp3-target genes in YY1-transduced T_reg_ cells (Fig. 8d,e) as well as in T_conv_ cells (Fig. 8f). Taken together, these data suggest that YY1 interacts with Foxp3, blocks binding of Foxp3 to its target genes and binds to the Foxp3-target genes, and that YY1 binding inhibits expression of these genes.

### Domains of YY1 critical for inhibition of T_reg_ functions

**Figure 9:**
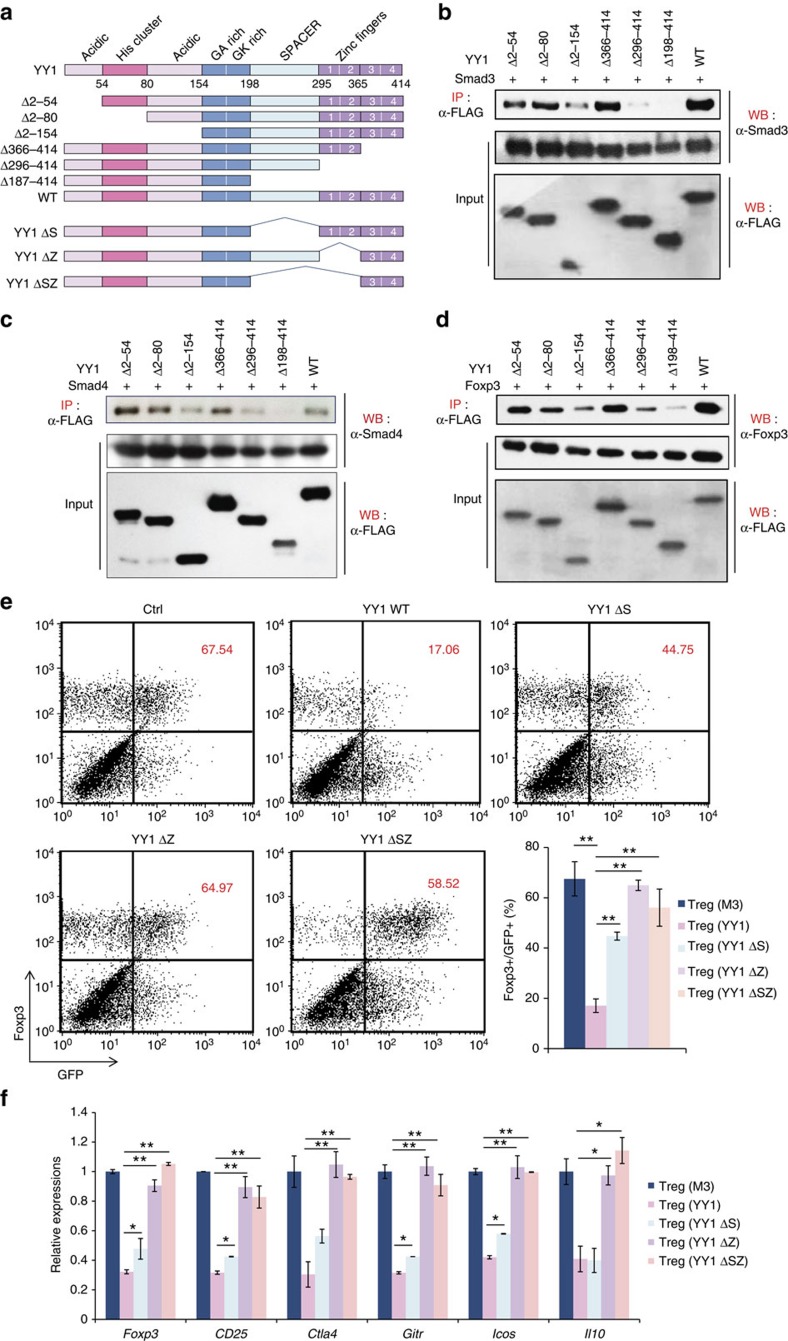
Spacer and zinc finger 1-2 domains of YY1 are essential for inhibition of T_reg_ differentiation. (**a**) Schematic diagram of YY1 domains. (**b**–**d**) HEK293T cells were transfected with Smad3-, Smad4- or Foxp3-expression vector together with various FLAG-tagged, YY1 domain mutant expression vectors. Cell lysates were immunoprecipitated with anti-FLAG. Then, proteins were immunoblotted by an anti-Smad3 (**b**), anti-Smad4 (**c**), anti-Foxp3 (**d**) or anti-FLAG antibody, as indicated. IP, immunoprecipitation; WB, immunoblot. (**e**) Naïve CD4 T cells were transduced with a retroviral vector containing control, YY1 (full length), YY1 ΔS, YY1 ΔZ or YY1 ΔSZ, and differentiated into T_reg_ cells for 4 days. Expression of Foxp3 was measured by flow cytometry. Numbers in the plots indicate percentage of Foxp3^+^ cells from GFP^+^ cells. (**f**) GFP^+^ cells from **e** were sorted, and total RNA was isolated. Relative amounts of the *Foxp3* and T_reg_ signature genes were measured by qRT–PCR. Experiments were performed three times with similar results. Error bars shown in **b** and **f** represent s.d. Statistical differences in **e** and **f** were analysed by Student's *t*-test (*n*=3). *P<0.05. **P<0.01.

To identify which domains of YY1 are important for interaction with other proteins, expression vectors containing YY1 deletion mutants were constructed (Fig. 9a). Protein–protein interactions between the YY1 deletion mutants and Smad3, Smad4 or Foxp3 were investigated using co-IP assays. YY1 was associated with Smad3 (Fig. 9b), Smad4 (Fig. 9c), Foxp3 (Fig. 9d) and Smad2 (Supplementary Fig. 5A). Deletion of the spacer or zinc finger 1-2 domains of YY1 caused loss of interactions with Foxp3, Smad3 and Smad4 (Fig. 9a–d, Supplementary Figs 4A–C), suggesting that these domains mediate interactions with other proteins.

**Figure 10:**
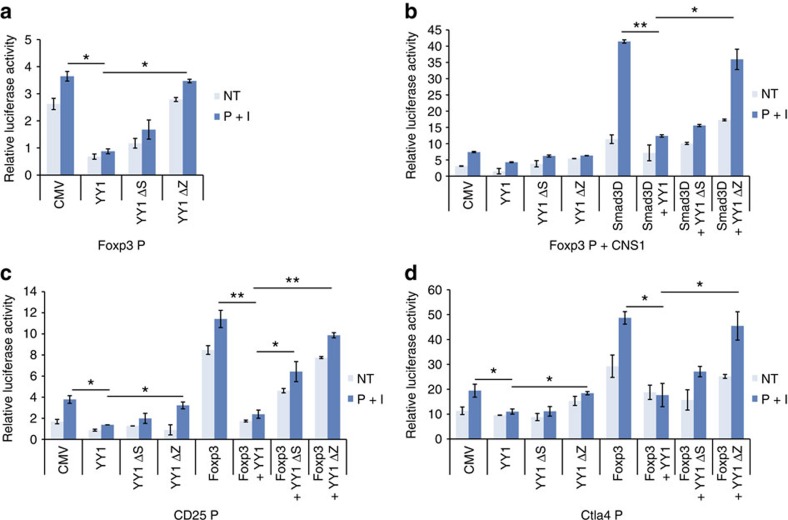
Spacer and Zinc finger domains of YY1 are essential for inhibiting transactivation activity of Smad3 or Foxp3. Transactivation activity of Smad3, Foxp3 or YY1 domain mutants at the promoters of the *Foxp3*, *CD25* or *Ctla4* genes was measured by transient reporter assay. EL4 cells were transfected with Foxp3 promoter-luc (**a**), Foxp3 promoter-CNS1-luc (**b**), CD25 promoter-luc (**c**) or Ctla4 promoter-luc (**d**) reporter construct in combination with *Smad3D*-, *Foxp3*- or *YY1* domain mutant expression vectors. Error bars shown in **a**–**d** represent s.d. Statistical differences were analysed by Student's *t*-test (*n*=3). *P<0.05. **P<0.01. I, ionomycin; NT, no treatment; P, phorbol 12-myristate 13-acetate.

To examine whether the spacer and zinc finger 1-2 domains of YY1 play an essential role in the inhibition of T_reg_ differentiation and function, we constructed retroviral vectors containing YY1 deletion mutants that have a deletion in spacer (YY1 ΔS) or zinc finger 1-2 domains (YY1 ΔZ), transduced them into naïve CD4 T cells, and differentiated them into T_reg_ cells. Foxp3 levels were greatly reduced in YY1-transduced T_reg_ cells compared with those in control T_reg_ cells (Fig. 9e). However, all the YY1 deletion mutants failed to reduce Foxp3 expression in T_reg_ cells (Fig. 9e). In addition, these mutants failed to decrease T_reg_ cell signature genes regulated by Foxp3 in T_reg_ cells (Fig. 9f). To further examine the role of YY1 deletion mutants in Foxp3-mediated target gene expression, a transient reporter assay using reporter constructs containing the promoter of Foxp3 (Foxp3P), Foxp3P-CNS1, CD25P or Ctla4P was performed in the presence of various YY1 deletion mutants. Full-length YY1 repressed the expression of *Foxp3*, *Cd25* and *Ctla4* genes mediated by Smad3D or by Foxp3 (Fig. 10). However, YY1 ΔZ failed to inhibit the transactivation of the *Foxp3* gene by Smad3D, suggesting that the inhibition of Foxp3 expression is mediated by the zinc finger 1-2 domains of YY1 (Fig. 10a,b). In addition, YY1 ΔS or YY1 ΔZ failed to inhibit transactivation of *Cd25* and *Ctla4* mediated by Foxp3 (Fig. 10c,d). These results strongly suggest that the spacer and zinc finger 1-2 domains of YY1 are critically important for its inhibition of Smad3 and Foxp3 functions.

## Discussion

The results of this study show that YY1 inhibits differentiation and function of T_reg_ cells. YY1 expression is lower in T_reg_ cells than in T_conv_ cells, and overexpression of YY1 reduces the expression of Foxp3 and its target genes. Furthermore, YY1 abrogates the immunosuppressive function of T_reg_ cells both *in vivo* and *in vitro*. YY1 directly binds to the *Foxp3* gene, reducing its expression, and represses chromatin remodelling of the locus. YY1 interacts with Smad3/4 and blocks their induction of *Foxp3* expression. Furthermore, YY1 inhibits the induction of Foxp3-target genes by binding to the Foxp3 protein and by blocking its binding to its target loci.

As Foxp3 is the critical transcription factor for the differentiation and maintenance of T_reg_ cells, understanding the molecular mechanisms for the regulation of Foxp3 is crucial for controlling immune homeostasis^3^,^11^,^12^. Although many factors have been shown to induce or stabilize Foxp3 (refs 3, 11, 12), only a few factors were shown to inhibit the expression or function of Foxp3. In some cases of diseases such as chronic infectious diseases and cancer, heightened T_reg_ function is an obstacle for complete cure of the diseases^7^. In these cases, negative regulation of Foxp3 function could help to boost protective immunity against these diseases. In this study, YY1 is characterized as a new negative regulator of Foxp3 and of T_reg_ cells.

In this study, we found that YY1 has pleiotropic functions and multiple roles in the inhibition of T_reg_ cell development and function (summarized in Supplementary Fig. 6). First, YY1 binds directly to regulatory regions in the *Foxp3* locus, which suppresses the expression of the *Foxp3* gene. Similar inhibitory mechanisms of regulation of the *Foxp3* gene were suggested in previous studies: GATA3 (ref. 14 and RORγt^16^ bind to the *Foxp3* promoter, and STAT3 (ref. 15) binds to the Foxp3 CNS2, which cause a repression of the *Foxp3* gene. How YY1 inhibits the *Foxp3* gene after binding to the *Foxp3* locus is not yet clear. One possibility is that YY1 may recruit chromatin remodelling factors to induce repressed chromatin status at the locus. YY1 interacts with many chromatin remodelling factors including the INO80 complex, histone acetyltransferases, histone deacetylases and histone methyltransferase^17^,^18^,^19^,^35^. The finding that YY1 reduced H3K4-me3 at the *Foxp3* locus supports this possibility; however, the detailed mechanisms of YY1-mediated repression of the *Foxp3* gene remain to be elucidated.

Second, YY1 inhibits the induction of *Foxp3* expression by blocking TGF-β signalling. A previous study showed that YY1 interacts with Smad proteins to inhibit TGF-β or BMP signalling^25^. The present study shows that YY1 has a similar role in inhibiting T_reg_ cell development. YY1 associates with Smad3 and Smad4 to block their binding to the Foxp3 CNS1, which is a crucial Smad-binding site for *Foxp3* expression during T_reg_ cell differentiation.

Third, YY1 inhibits Foxp3-induced expression of target genes by physically binding to and blocking Foxp3. Foxp3 is a global regulator of its target genes; Foxp3 binds to numerous target genes and induces their expression, which is essential for T_reg_ cell differentiation and function. Previous studies have shown that Foxp3 is capable of binding several proteins^24^. For example, Foxp3 binds to AML1 and inhibits the production of the cytokine IL-2 (ref. 36). Foxp3 also binds to NFAT and interferes with the conformation of AP1–NFAT complexes that are essential for effector cell programmes^37^. Foxp3 also interacts with GATA3 (refs 24, 38) and RORγt^39^, and these interactions lead to inactivation of Foxp3. Our data show that YY1 binds to Foxp3 and abrogates its function. YY1-mediated inhibition of the Foxp3-target genes can be explained by two possible mechanisms. The interaction of YY1 with Foxp3 may physically interfere with Foxp3 binding to its target genes. Another possibility is that YY1 binding to the Foxp3 target gene loci may inhibit Foxp3 binding to the loci. Here, YY1 directly bound to the promoter of the Foxp3 target genes. Although the cognate YY1-binding sites are separate from the Foxp3-binding site, YY1-binding sites appear to be present in the promoters of the Foxp3 target genes, including *Cd25*, *Icos*, *Ctla4* and *Gitr*.

Like other transcription factors interacting with Foxp3, such as RORγt, STAT3 and GATA3, YY1 is implicated in effector CD4 T-cell differentiation. We previously have shown that YY1 plays an important role in Th2-cell differentiation^23^. The set of transcription factors including YY1 have dual roles in CD4 T-cell differentiation: one is to stimulate a specific effector subset differentiation, and the other is to inhibit T_reg_-cell differentiation. Thus, the outcome of the reciprocal inhibition would be the fate decision of effector versus T_reg_-cell differentiation. Although most of these proteins are normally expressed exclusively with Foxp3, they may be co-expressed in certain situations such as during the course of infection. The proinflammatory response predominates at the initial phase of infection, whereas the anti-inflammatory response predominates at the later resolving phase of infection^40^. Reciprocal regulation of the effector-specific transcription factors and Foxp3 may determine proinflammatory versus anti-inflammatory reactions.

In conclusion, the present study illustrates that YY1 inhibits the differentiation and function of T_reg_ cells by blocking Foxp3 expression and function. This study elucidates fundamental molecular mechanisms of T_reg_ differentiation, and may contribute to the development of therapeutic strategies for many immune-related diseases and cancer.

## Methods

### Mice

Six- to eight-week-old C57BL/6 mice were purchased from Samtaco, and CD4-CRE transgenic mice were purchased from Taconic. RAG1-deficient (C57BL/6) mice, YY1 flox/flox (C57BL/6) mice, Foxp3-eGFP (C57/BL6) and Foxp3-RFP (C57/BL6) mice were purchased from Jackson Laboratory. YY1 KD mice were previously described^23^. All mice were maintained in the Sogang University animal facility under specific pathogen-free conditions. Experiments with live mice were approved by the Sogang University Institutional Animal Care and Use Committee.

### *In vitro* differentiation of CD4 T cells

CD4 T cells were enriched from spleen cells from WT mice by mixing with anti-MHC class II (M5/115, cat. no. MABF33, Millipore, diluted 1/200), anti-NK1.1 (PK136, cat. no. 108712, BioLegend, diluted in 1/200) and anti-CD8 (53-6.7, cat. no. 100735, BioLegend, diluted in 1/200) antibodies, followed by depletion with a mixture of magnetic beads conjugated to anti-rat IgG (BioMag, cat. no. 84334, Polybioscience, diluted in 1/10) and anti-mouse IgG antibodies (BioMag, cat. no. 84340, diluted in 1/10). Naïve CD4 T cells were sorted based on the surface markers, CD4^high^, CD62L^high^ and CD44^low^. The cells were activated by plate-bound anti-CD3ɛ (2C11, BioLegend, 5 μg ml^−1^) and soluble anti-CD28 (37.51, BioLegend, 2 μg ml^−1^) antibodies. For neutral (Th0) differentiation, 1 × 10^6^ naïve CD4 T cells were cultured in 5 ml of RPMI-1640 culture medium (Life Technologies) supplemented with 5% fetal bovine serum, 2-mercaptoethanol, MEM amino acids solution (Life Technologies), non-essential MEM amino acids solution (Life Technologies) and penicillin–streptomycin solution (Life Technologies) in the presence of IL-2 (1 ng ml^−1^). For Th1 skewing conditions, IL-12 (3.5 ng ml^−1^) and 11B11 (anti-IL-4, 10 μg ml^−1^) antibody were added to same media. For Th2 cell differentiation, IL-4 (5 ng ml^−1^) and XMG1.2 (anti-IFN-γ, 10 μg ml^−1^) antibody were added. T_reg_ conditions comprised treatment with TGF-β (5 μg ml^−1^), IL-2 (1 ng ml^−1^), XMG1.2 antibody (10 μg ml^−1^) and 11B11 antibody (10 μg ml^−1^). After 4–5 days, the cells were used for further experiments.

### EMSA

Nuclear extracts (5 μg) were incubated for 30 min on ice in binding buffer (10 mM Tris-Cl, pH 8.0, 40 mM KCl, 0.05% NP-40, 6% glycerol, 1 mM dithiothreitol (DTT), 1 μg μl^−1^ of poly(dI:dC)) and then 0.5 ng of ^32^P-labelled double-stranded oligonucleotides were added to the mixture and incubated for 30 min on ice. The oligonucleotide sequences used are described in Supplementary Table 1.

### Chromatin immunoprecipitation

Cells (1 × 10^7^) were cross-linked with 1% paraformaldehyde on ice for 30 min and quenched with 0.125 M glycine. Cells were then lysed with a buffer containing 1% SDS and sonicated at the high-power setting for 15 min using a Bioruptor sonicator (Diagenode). Cell extracts were pre-cleared with protein A/G agarose/salmon sperm DNA (Upstate) and incubated with an anti-H3K4-me3 (Millipore, 07-473), anti-H3Ac (Millipore, 06-599), anti-YY1 (Santa Cruz, sc-1703), anti-FLAG (Sigma, M2) or normal rabbit IgG or normal mouse IgG (Santa Cruz) as a negative control. Antibody-bound protein–chromatin complexes were precipitated by protein A/G agarose, washed and eluted. The chromatin was reverse cross-linked by incubating at 65 °C for 4 h, followed by protease K treatment. After clean-up, the amount of precipitated DNA was quantified by quantitative PCR using the SYBR green (Kappa Bio) method with the primers listed in Supplementary Table 2.

### RNA isolation and qRT–PCR

Total RNA was isolated from naive CD4 T cells or *in vitro*-differentiated Th0, Th1, Th2 or T_reg_ cells using Trizol reagent (Invitrogen). Reverse transcription (RT) was performed using Superscript II RT (Topscript). Quantitative PCRs for cDNA were performed with real-time fluorogenic 5′-nuclease PCR or SYGR green method using the 7500 Real-Time PCR System (Applied Biosystems). Relative amounts of expression were normalized by the amount of the *Hprt* transcript. The dual labelled probe and primer sequences used for quantitative PCR are listed in Supplementary Table 3.

### Microarray analysis

The synthesis of target cRNA probes and hybridization were performed using Agilent's Low RNA Input Linear Amplification kit (Agilent Technologies) according to the manufacturer's instructions. Briefly, each 1 μg total RNA and T7 promoter primer mix and incubated at 65 °C for 10 min. cDNA master mix (5 × First strand buffer, 0.1 M DTT, 10 mM dNTP mix, RNase-Out and MMLV-RT) was prepared and added to the reaction mixer. The samples were incubated at 40 °C for 2 h and then the RT and double-stranded (dsDNA) synthesis was terminated by incubating at 65 °C for 15 min. The transcription master mix was prepared as the manufacturer's protocol (4 × Transcription buffer, 0.1M DTT, NTP mix, 50% PEG, RNase-Out, Inorganic pyrophosphatase, T7-RNA polymerase and Cyanine 3/5-CTP). Transcription of dsDNA was performed by adding the transcription master mix to the dsDNA reaction samples and incubating at 40 °C for 2 h. Amplified and labelled cRNA was purified on cRNA Cleanup Module (Agilent Technologies) according to the manufacturer's protocol. Labelled cRNA target was quantified using ND-1000 spectrophotometer (NanoDrop Technologies). After checking labelling efficiency, fragmentation of cRNA was performed by adding 10 × blocking agent and 25 × fragmentation buffer and incubating at 60 °C for 30 min. The fragmented cRNA was resuspended with 2 × hybridization buffer and directly pipetted onto assembled Agilent's Mouse Oligo Microarray (60 K). The arrays hybridized at 65 °C for 17 h using Agilent Hybridization oven (Agilent Technologies). The hybridized microarrays were washed as the manufacturer's washing protocol (Agilent Technologies). The hybridized images were scanned using Agilent's DNA microarray scanner and quantified with Feature Extraction Software (Agilent Technologies). All data normalization and selection of fold-changed genes were performed using GeneSpringGX 7.3 (Agilent Technologies). Functional annotation of genes was performed according to the Gene OntologyTM Consortium (http://www.geneontology.org/index.shtml) by GeneSpringGX 7.3 (Agilent Technologies). Gene classification was based on searches done by BioCarta (http://www.biocarta.com/), GenMAPP (http://www.genmapp.org/), DAVID (http://david.abcc.ncifcrf.gov/) and Medline databases (http://www.ncbi.nlm.nih.gov/). The microarray data were deposited in GEO database (GEO accession number: GSE75052).

### Immunoblot analysis

Protein or cell extracts were resolved on a 10% SDS–PAGE gel and transferred to a polyvinylidene difluoride membrane (Bio-Rad). The membrane was blocked with 5% skim milk in TBST for 1 h at room temperature. The membrane was then probed with an antibody against FLAG, Foxp3 (eBioscience), Smad2 (Cell Signaling, 5339), pSmad3 (Cell Signaling, 9520), Smad3 (Abcam, 29379), Smad4 (Cell Signaling, 9515), YY1 (Santa Cruz, sc-1703) or β-actin (Santa Cruz, sc-47778), diluted 1:100 or 1:1,000 in TBST overnight at 4 °C. An HRP-conjugated antibody against rabbit or mouse (BioLegend) diluted at 1:2,000 in 5% skim milk TBST was added for 1 h at room temperature. Target proteins were detected by enhanced chemiluminescence reaction. Images have been cropped for presentation. Full-size images are presented in Supplementary Fig. 7.

### DNA pull-down assay

Nuclear extracts were isolated from HEK293T cells by sonication. Chromatin DNA was pelleted at 14,000*g* for 15 min at 4 °C, and the nuclear lysates in the supernatant were collected. Biotin-labelled DNA probes (1 μg) were incubated with nuclear lysate with proteinase inhibitors for 30 min at room temperature in binding buffer (10 mM Tris-Cl, pH 8.0, 50 mM KCl, 1 mM DTT, 5% glycerol, 1 μg ml^−1^ poly(dI:dC), 1 mg ml^−1^ salmon sperm DNA, 1 mg ml^−1^ BSA). Protein–DNA complexes were collected with streptavidin agarose (50 μl, Sigma-Aldrich). Precipitated proteins were washed five times with binding buffer and resuspended in an SDS loading buffer. Immunoblot analysis was performed as described above.

### Retroviral transduction

For ectopic expression, mouse *Yy1* (WT or deletion mutants) coding sequences were cloned into MIEG3 retroviral vector. A total of 1 × 10^6^ Phoenix Eco cells were co-transfected with retroviral vector and pCL-Eco helper vector. A culture supernatant containing high titres of retrovirus was collected after 48 h of transfection. Purified naive CD4 T cells were activated under Th0 condition with plate-bound anti-CD3 (5 μg ml^−1^) and anti-CD28 (2 μg ml^−1^) for 24 h. Activated cells were then spin-infected in 1 ml of retrovirus-containing supernatant with polybrene (4 μg ml^−1^) at 1,500*g* for 90 min at 32 °C. After the spin infection, cells were incubated for 4–5 days under the Th0 or T_reg_ condition. GFP^+^ cells or total cells were used for further experiments.

### FACS staining

For Foxp3, cells were stained with anti-Foxp3 (eBioscience) using a Foxp3 staining kit (BioLegend). For YY1 staining, total splenocytes from YY1-eGFP mice were enriched by CD4 microbeads (Miltenyi). T_conv_ (CD4^+^CD25^−^) or T_reg_ (CD4^+^ CD25^+^) cells from WT mice were prepared using a CD4^+^CD25^+^ T_reg_ isolation kit (Miltenyi). The cells were stained with an anti-YY1 (Santa Cruz sc-1703) antibody using a Foxp3 staining kit (BioLegend). For cytokine staining, cells were restimulated with 1 μM ionomycin (Sigma-Aldrich) and 10 nM phorbol myristate acetate (Sigma-Aldrich) with Golgi stop (BD Bioscience) for 4 h. Intracellular staining was performed using Cytofix/cytoperm kit (BD Bioscience).

### Co-immunoprecipitation

HEK293T cells were transfected with FLAG-tagged pCMV-*Yy1*, pCMV-*Smad3*, pCMV-*Smad4* and pCMV-*Foxp3*. Two days after transfection, cell lysates were isolated and then pre-cleared with control IgG followed by protein A/G (Santa Cruz) treatment. Pre-cleared lysates were incubated overnight at 4 °C with anti-YY1, anti-Smad3, anti-Smad4, anti-Foxp3, anti-FLAG or control IgG antibody. Then, protein A/G beads were added, followed by incubation for an additional 4 h. Immunocomplexes were extensively washed and resuspended in an SDS loading buffer. Immunoblot analysis was performed as described above.

### Dual luciferase assay

EL4, a mouse lymphoma cell line, was co-transfected with a combination of CMV-*Yy1*, CMV-*Smad3D* or CMV-*Foxp3* expression vectors, and pGL-*Foxp3*P or pGL-*Foxp3* P/CNS1, pGL-*Il2ra*P, pGL-*Icos*P, pGL-*Ctla4*P or pGL-*Gitr*P reporter vectors. The following day, luciferase activity was measured. Transfection efficiency was normalized by dividing *Firefly* luciferase activity by *Renilla* luciferase activity.

### *In vitro* suppression assays of YY1-transduced T_reg_ cells

T_reg_ cells were isolated from Foxp3-RFP knock-in mice based on RFP expression. Isolated T_reg_ cells were transduced with control or YY-1-expressing retroviral vector as described above. After sorting of GFP^+^-transduced T_reg_ cells, 1 × 10^5^ cells were plated in 96-well plates. CD4^+^CD25^−^ T_resp_ cells were freshly isolated from CD45.1 mice on C57BL/6 background, and were labelled with CFSE (Sigma). T_resp_ cells (1 × 10^5^) were added together with anti-CD3/CD28 beads (Invitrogen) in 96-well plates. After 3 day cultures, T_resp_ cells were selected and analysed using FACS caliber (BD Bioscience).

### Induction of inflammatory bowel disease

Naïve CD4 T (CD4+CD62^hi^CD45RB^hi^) cells from C57BL/6 mice were sorted on a FACS Aria III (BD Bioscience). Inflammatory bowel disease was induced by the adoptive transfer of 5 × 10^5^ naïve CD4 T cells into *Rag*1−/− mice by retro-orbital injection. Naïve CD4 T cells (5 × 10^5^) alone or together with control vector-transduced GFP^+^ T_reg_ cells (1 × 10^5^) or YY1-transduced GFP^+^ T_reg_ cells (1 × 10^5^) were transferred into *Rag*1−/− mice. Recipient mice were weighed two times per week. After 9 weeks, mice were killed, and splenocytes and CD4 T cells were counted by flow cytometry. For histological analysis, the colon was removed and fixed in 10% (vol/vol) buffered formalin solution, and sections were stained with haematoxylin and eosin.

### Statistical analysis

Values are shown as the mean±standard deviation (s.d.). Statistical differences between mean values were determined by Student's *t*-test. Results were considered significant when the *P*-value was less than 0.05.

## Additional information

**How to cite this article:** Hwang, S. S. *et al.* YY1 inhibits differentiation and function of regulatory T cells by blocking Foxp3 expression and activity. *Nat. Commun.* 7:10789 doi: 10.1038/ncomms10789 (2016).

## Supplementary Material

Supplementary Information Supplementary Figures 1-7 and Supplementary Tables 1-3

Supplementary Data 1 Microarray data: spread sheet
