# Supplementary material for: YY1 inhibits differentiation and function of regulatory T cells by blocking Foxp3 expression and activity
Source: Nat Commun. 2016 Feb 19;7:10789. doi: 10.1038/ncomms10789 (PMC4762897; doi:10.1038/ncomms10789)
Supplement: Supplementary Information — Supplementary Figures 1-7 and Supplementary Tables 1-3 [file ncomms10789-s1.pdf]

Supplementary Figures

A

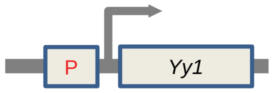

Promoter

AAATCTAAGAAACAAATAAGATACACTTGGCTCAAATTTTCG
GTTCTTGGAAAAAAAAAAAGGCAATCTAACTTTATGTTGCTTT
AAACCACACCTGGTCTATCGAAAGGAAGCACAGAAGAAACA
CTTCACTACAATGTATCTTAAGCAATTTGATGATTTGAACAAG
GAAAACCTAAGCTGTTACTTATAATAGGGAAATCACAAAAGA
CATGGAATTTTCAAAGAGCTAAATGCAAAGACTATTTAAAAG
GAATATTTTTAAATAGTCCTTTTCGGCAAAAGGAATGTTTATG
AGCGAGGAATAGGCGAAGC

Foxp3 site-1 (-1498) : AATCTAAGAAACAAATAAGATACACT
Foxp3 site-2 (-1217) : GCAAAAGGAATGTTTATGAGCGAGGA

B

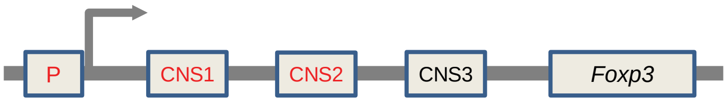

Promoter

TCTGGGAGCCAGCCATTCTGAGACTCTCTGATTCTGTGAATT
TGTGGGGGG AGTACAGCCCACTTTTTTCTCCATGAATTGCTT
TCCATGCCTCTTGCCTTCTGTGGAAAGAAAGGCTACAGGAGT
GGCCAGCTCTGCCAAGCCTTGGCAACATGATGGTGGTGATC
ATATGC

YBS-1 : GGAGCCAGCCATTCTGAGA
YBS-2 : TTTCTCCATGAATTGCTTTCCATGCCT
YBS-3 : GGCAACATGATGGTGGTGAT

CNS1

ATGAGATAACTGTTCACCCCATGTTGGCTTCCAGTCTCCTTTAT
GGCTTCATTTTTTCCATTTACTGCAGAGGTCAAAAGTGTGGGT
ATGGGAGCCAGACTGTCTGGAACAACCTAGCCTCAACTCAAG
TCATCTGTGTGAATTTTACCCAGGCTCTTAACCTCTCTGTACCT
CCATTTCCTCGTATGTACTGTGATGAT

YBS-4 : CTGTTCACCCCATGTTGGCTTC
YBS-5 : TCCTTTATGGCTTCATTTTTTCCATTTACTGCA
YBS-6 : TCTGTACCTCCATTTCCTCGT

SBE : GGTATGGGAGCCAGACTGTCTGGAACAACCTA

CNS2

CCGATGAAGCCCAATGCATCCGGCCGCCATGACGTCAATGGCA
GAAAAATCTGGCCAAGTTCAGGTTGTGACAAC

YBS-7 : ATCCGGCCGCCATGACGTCAATGGCAGAAA

C

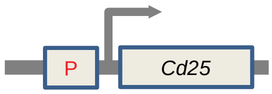

Promoter

GCAGGTTTGAAACAAAACTTGGACATGTGCTCACAAGCATT
TGTCTGAAATGGCTTGAAATGTATCCTTGTTGTATATTCACAT
TCACATATCTCAATGCATCATGGTTGAACACCCATATGTGTTA
TGAAATCAGTGCTTATTTCAAAAATATATTGGATGTAGCCTGT
CTCCAGAGCCCACAGTGTGATACCTTTATCCTAAGATCCTCA
GACTCTTATGAGTTCAAAGTGCCTGTGACACTCCTGTAACAC
TCCTGTCTATAAATATACTGATTATATGCTGGCGTGCATTTTTC
CCTCAAGGGAGGAAATTAAGATTTCACCAGATCTTATTTGCA
AAACAAAACAGTCAGCAACTAGGCTAGAAGTAAAAAGTTATG
TCAATAGTGGGTGAAAAGACAGCTTGGTGACACTATGAGAG
AAGGCAAAGGGTTCTTTCTGAAAGGCCTCCTGGGAGAAGCT
AGACTTAAAATCTTCCATTGCAGCTGTAAACACATCTGGACA

YBS(-975) :
ATGCATCATGGTTGAACACCCATATGTGT

D

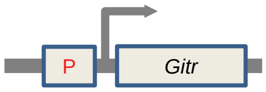

Promoter

GAGGAGGAAATGGCCAAATAAGAGAAAAGACCTTGGTGGG
CAGGTAACCCTGAGTTTAGGGGTGTAATCTAGCCAGTCAGA
GAGAATGGAAGAGAAGGGCAAGGCCTGTCAGAGCCATGCT
GGCCATGTTCAAGAAAATGAGGCTCACCTGTATCTGGGACT
CCTTTCTTGTTGTCTAAAATTGTGTGAGGAAGTGAACAAAAG
TGGCTCCAGGGAGAGCCAAGCTGGAACTGCCAAGGCTAGA
CCTGGGCAGGCCACACCGCCCATGGCTTCATGTACTTGCTA
GGAATACCTACTTTATGATGATCTTCTTAGCGAGGCATGGTG
TGTGCTCTGGACTCCTCTCGCTGGCTCTCCTAGTTTCCCAG
GCTTAGAGGAACTTCCTGGGGCAGAAGGGGGAAGGGGGTA
GTTTTATACAATAAGGGGTGCCCACAGGTGGCAGCAGTGGC
ACATGTGAATGGGCATCCTTTTCCTCTGTAGCCTAGCCAGG
GCGAGTTTGCTGGAGTTGGGAGAGGCATGTAGGGGTTAGG
AGATGTCCAGAAAGGGGATAGCTCTGCTCTACACTTCACAG
AAGGCCTTTACAAACACCTCAGATGT

YBS (-581) :
GTCAGAGCCATGCTGGCCATGTTCAAG

E

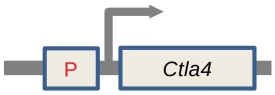

Promoter

CAGGACTAAGAGGGATGGCCATGGGACGCTAAGTAAGA
GTACTGGGGGATTAAAGATGACCAGATGACTGGATAAAG
TTGGGACTGGGATTTAGGGATTCCTTGTACTACAGAAATT
ATACTCTCCAAGACTCCACGTCTCCAGGTCCTCAGAGGT
GACTCGAAGCTTCAGTTTCAAGTTGAGTACATTTTCCATC
CATGGATTTGCTTGTTTTGTTCAGTTTTAGTTTGAATATTT
GAGGTCGTCTTTACGACGTAACAGCTAAACCCACGGCTT
CCTTTCTCGTAAAACCAAAACAAAAAGGCTCTCTGTTCAG
GTGTCCTGTGTGTGCA

YBS(-318) : AAGAGGGATGGCCATGGGACGCT

Supplementary Figure 1. Sequences in the regulatory elements of the *Yy1*, *Foxp3*, *Cd25*, *Gitr*, and *Ctla4* genes

(A-E) DNA Sequences in the regulatory elements of the *Yy1* **(A)**, *Foxp3* **(B)**, *Cd25* **(C)**, *Gitr* **(D)**, *and Ctla4* **(E)** genes. Oligomers used in EMSA were as indicated. Red characters mean putative YY1 binding sites. Blue characters indicate putative Foxp3 binding sites. Green characters indicate Smad-binding elements. P, promoter; CNS, conserved non-coding sequence.

A

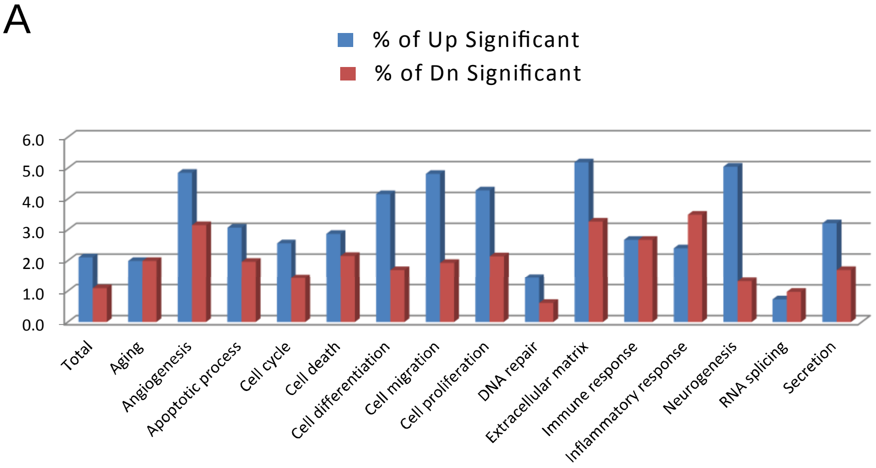

B

gene	YY1/cont
Tgfb1	1.492
Gimap5	1.316
H2-M3	1.240
Il2rg	0.980
Tnfrsf4	0.954
Irf1	0.908
Icos	0.870
Ctla4	0.817
Rltpr	0.722
Il2ra	0.687
Rltpr	0.685
Foxp3	0.664
Ctla2a	0.614
Tnfrsf18	0.606
Tnfsf4	0.495

Supplementary Figure 2. Microarray analysis of gene expression by YY1 overexpression in T_{reg} cells

A. Genes upregulated or downregulated more than two fold by YY1 overexpression were categorized by their cellular function. B. Differentially expressed genes by YY1 in a category of Treg cell differentiation.

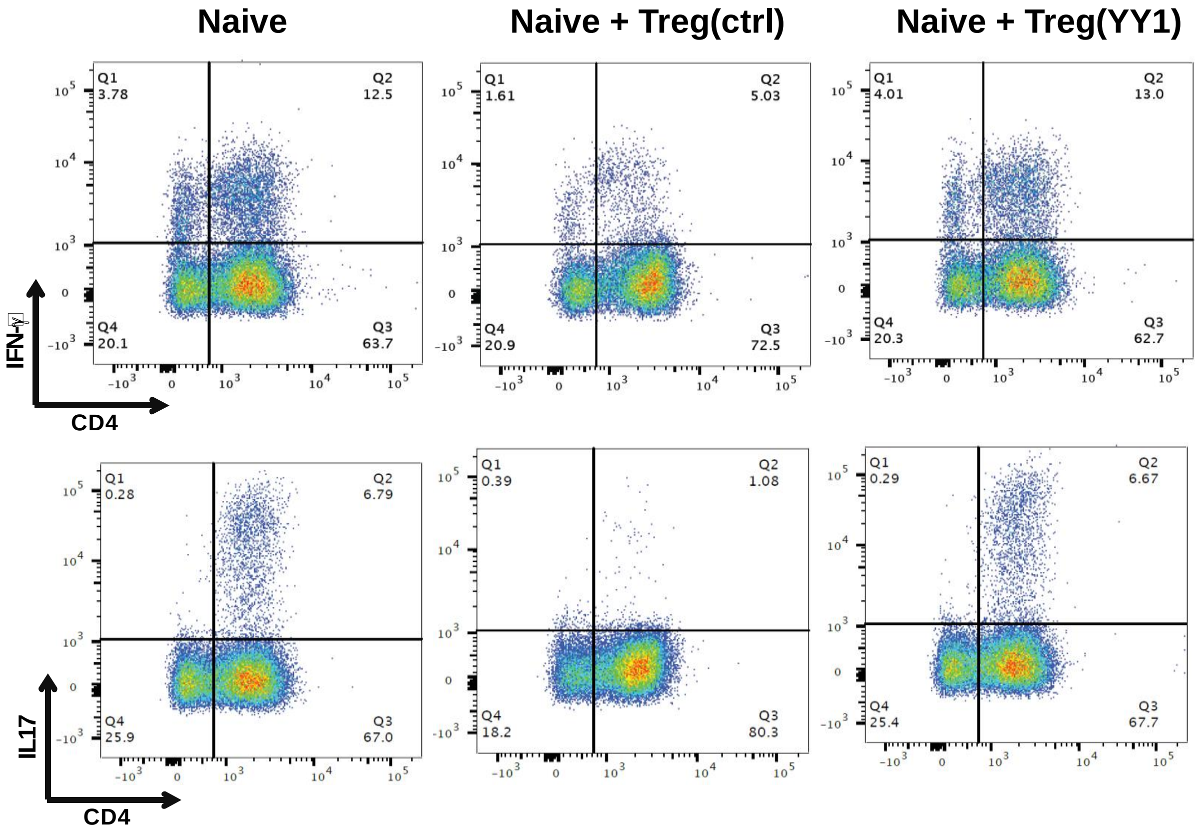

Supplementary Figure 3. YY1 impairs immunosuppressive function of T_{reg} cells *in vivo*

FACS plot of Figure 4F.

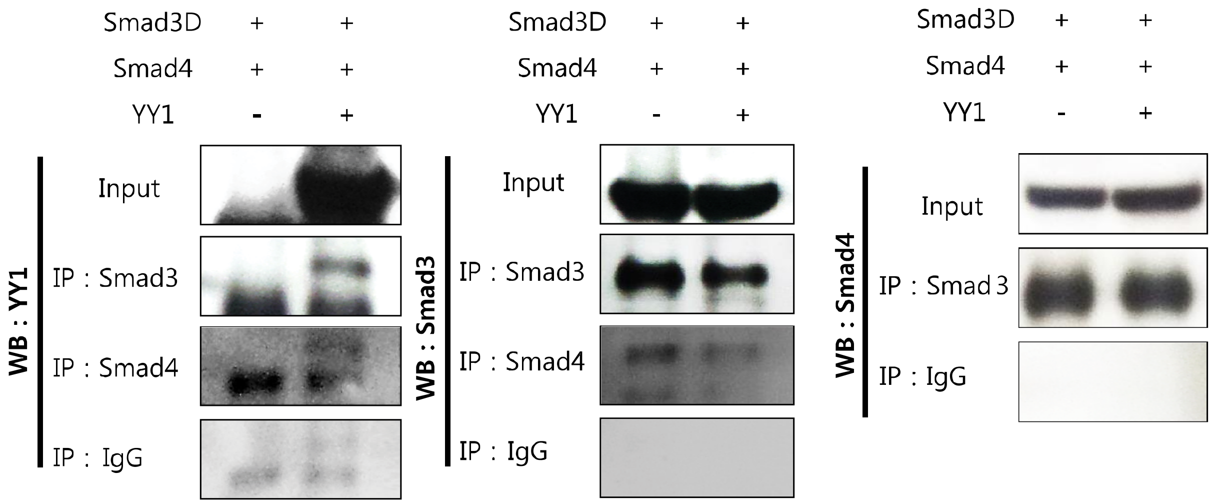

Supplementary Figure 4. YY1 does not interfere with the dimerization and translocation to the nucleus of Smad3/4

HEK293T cells were transfected with a *Smad3-, Smad4-*, or *Yy1*-expression vector. Cell lysates were immunoprecipitated with an anti-Smad3, anti-Smad4, or control IgG antibody. Then, proteins were immunoblotted by an anti-Smad3, anti-Smad4, or anti-YY1 antibody as indicated.

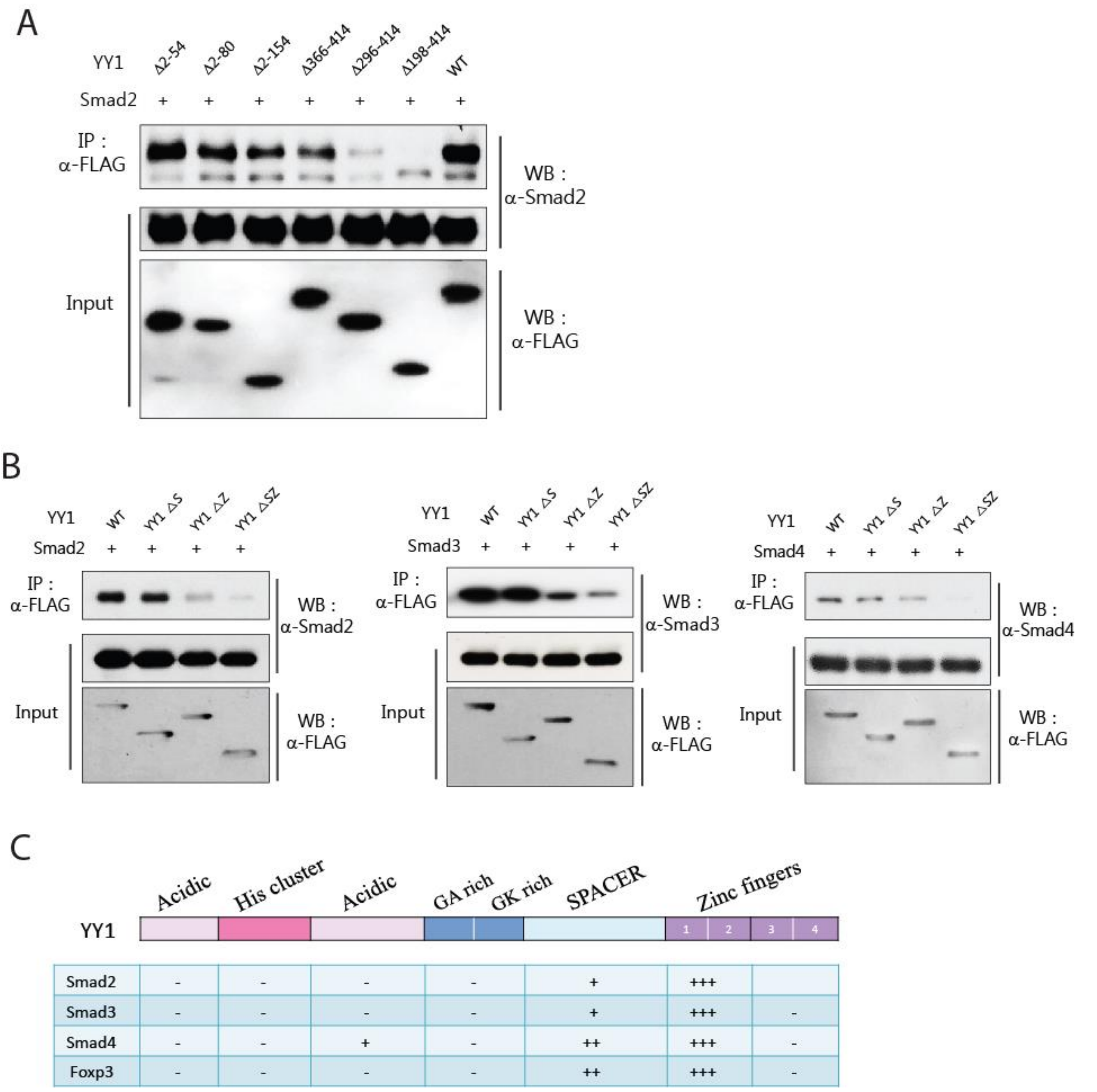

YY1	Acidic	His cluster	Acidic	GA rich / GK rich	SPACER	Zinc fingers 1–2	Zinc fingers 3–4
Smad2	-	-	-	-	+	+++	
Smad3	-	-	-	-	+	+++	-
Smad4	-	-	+	-	++	+++	-
Foxp3	-	-	-	-	++	+++	-

Supplementary Figure 5. Spacer and zinc finger domains of YY1 are essential for interactions with Smad and Foxp3

(A-B) HEK293T cells were transfected with a *Smad2*- and various FLAG-tagged, *Yy1* domain mutants-expression vectors. Cell lysates were immunoprecipitated with an anti-FLAG antibody. Then, the proteins were immunoblotted by an anti-Smad2 **(A and B)**, anti-Smad3 **(B)**, anti-Smad4 **(B)**, or anti-FLAG antibody as indicated. **(C)** Summary of YY1 domain binding affinity.

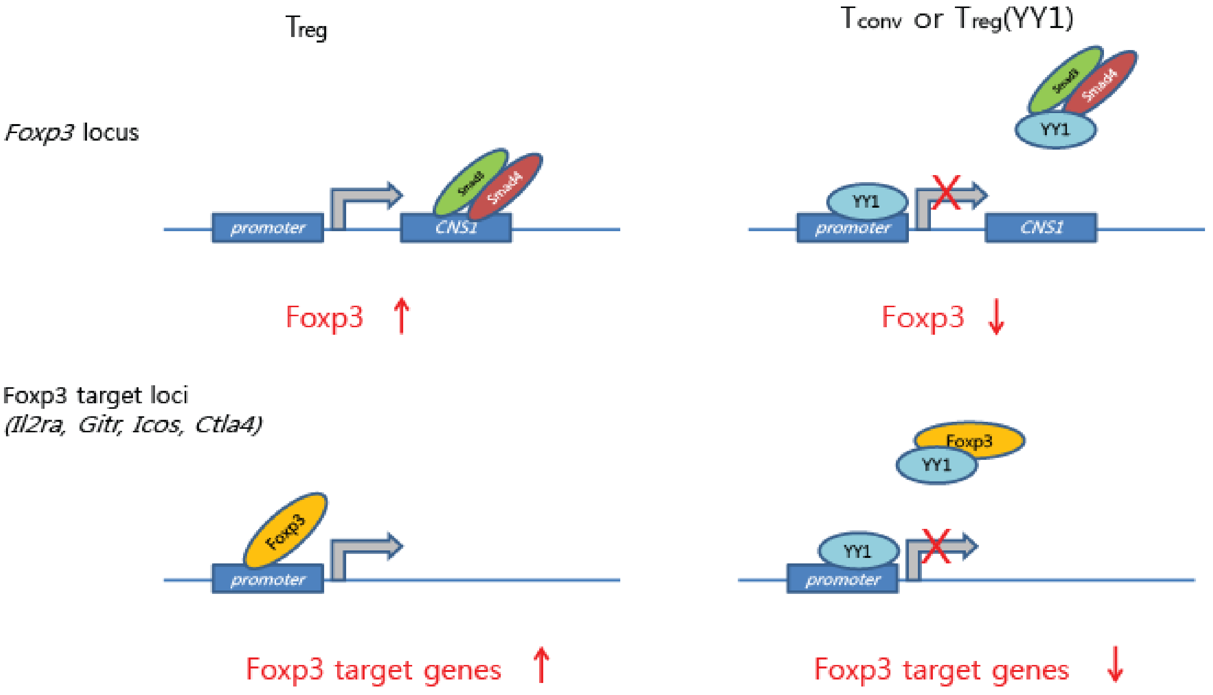

Supplementary Figure 6. A model for the YY1-mediated inhibition of T_{reg} differentiation

Proposed mechanisms for YY1-mediated inhibition of T_{reg} differentiation and function. First, YY1 inhibits expression of the *Foxp3* gene by binding directly to the *Foxp3* locus and by blocking Smad3/4 binding to the Foxp3 CNS1 in T_{conv} or YY1-overexpressing T_{reg} cells. Second, YY1 inhibits the expression of the Foxp3-target genes by blocking Foxp3 binding to its target genes and by competing with Foxp3 for binding to the promoters of the genes.

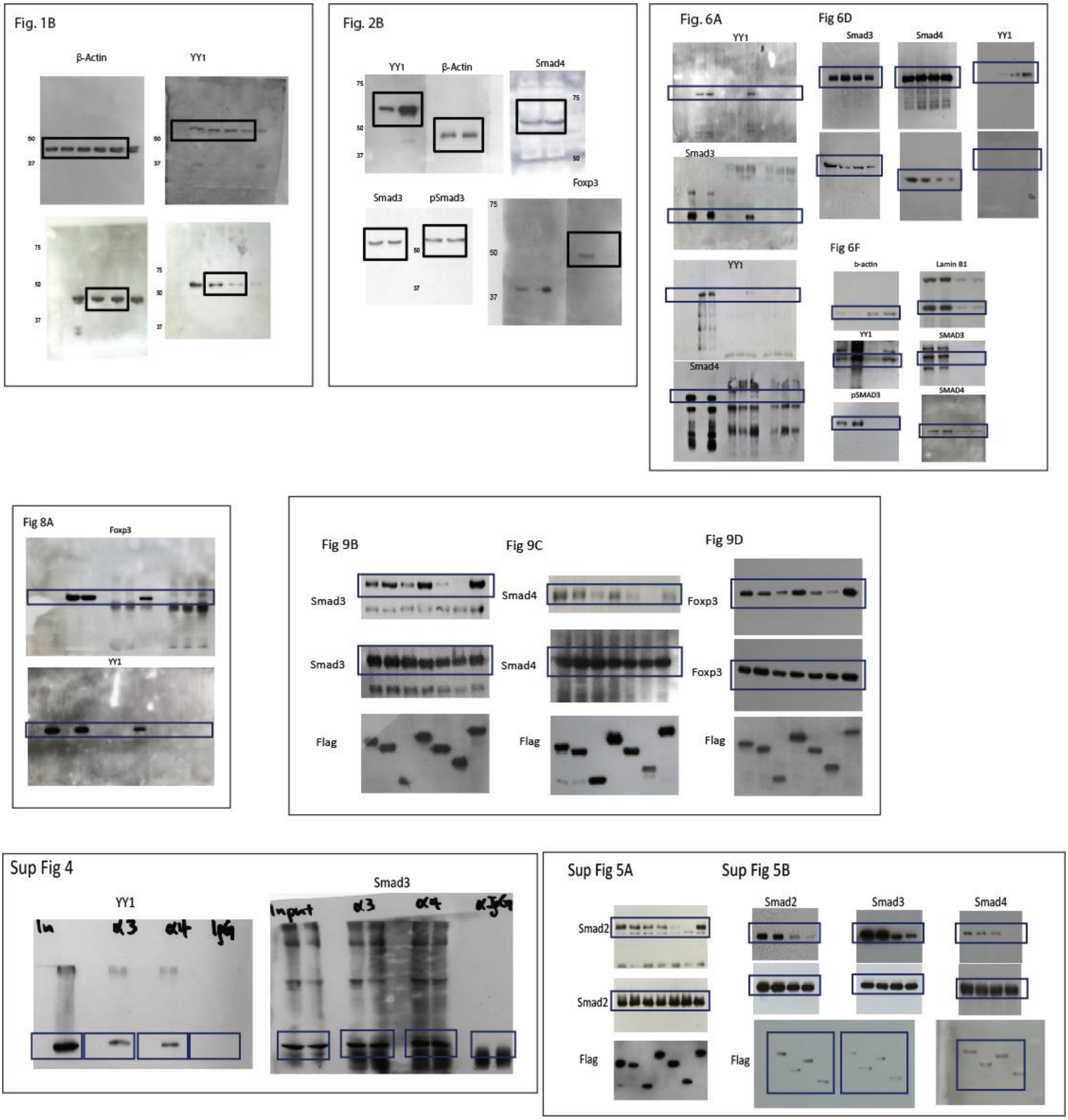

Supplementary Figure 7. Uncropped western blots of Figure 1, 2, 6, 8, 9, and Supplementary Fig. 4 and 5.

Supplementary Tables

Supplementary Table 1. Oligonucleotides used in EMSA

Primer name	Sequences (5'→3')
YY1 consensus F	CGCTCCCCGGCCATCTTGGCGGCTGGT
YY1 consensus R	ACCAGCCGCCAAGATGGCCGGGGAGCG
Foxp3 CNS1 SBE F	GGTATGGGAGCCAGACTGTCTGGAACAACCTA
Foxp3 CNS1 SBE R	TAGGTTGTTCCAGACAGTCTGGCTCCCATACC
Forkhead consensus F	AAGCGGTAAACAATGAATAAACAACGCGAT
Forkhead consensus R	ATCGCGTTGTTTATTCATTGTTTACCGCTT
Foxp3 CNS1-YBS1 F	CTGTTCACCCCATGTTGGCTTC
Foxp3 CNS1-YBS1 R	GAAGCCAACATGGGGTGAACAG
Foxp3 CNS1-YBS2 F	TCCTTTATGGCTTCATTTTTTCCATTTACTGCA
Foxp3 CNS1-YBS2 R	TGCAGTAAATGGAAAAAATGAAGCCATAAAGGA
Foxp3 CNS1-YBS3 F	TCTGTACCTCCATTTCCTCGT
Foxp3 CNS1-YBS3 R	ACGAGGAAATGGAGGTACAGA
Foxp3 CNS2-YBS F	ATCCGGCCGCCATGACGTCAATGGCAGAAA
Foxp3 CNS2-YBS R	TTTCTGCCATTGACGTCATGGCGGCCGGAT
Foxp3 promoter-YBS1 F	GGAGCCAGCCATTCTGAGA
Foxp3 promoter-YBS1 R	TCTCAGAATGGCTGGCTCC
Foxp3 promoter-YBS2 F	TTTTCTCCATGAATTGCTTTCCATGCCTC
Foxp3 promoter-YBS2 R	GAGGCATGGAAAGCAATTCATGGAGAAAA
Foxp3 promoter-YBS3 F	GGCAACATGATGGTGGTGAT
Foxp3 promoter-YBS3 R	ATCACCACCATCATGTTGCC
CD25 promoter-YBS F	ATGCATCATGGTTGAACACCCATATGTGT
CD25 promoter-YBS R	ACACATATGGGTGTTCAACCATGATGCAT
Gitr promoter-YBS F	GTCAGAGCCATGCTGGCCATGTTCAAG
Gitr promoter-YBS R	CTTGAACATGGCCAGCATGGCTCTGAC
Ctla4 promoter-YBS F	AAGAGGGATGGCCATGGGACGCT
Ctla4 promoter-YBS R	AGCGTCCCATGGCCATCCCTCTT

Supplementary Table 2. Primers used in ChIP assay

Primer name	Sequences (5'→3')
Foxp3 Promoter F	ATCCTCCAACGTCTCACAAACACA
Foxp3 Promoter R	TAACAGGGCTCATGAGAAACCACA
Foxp3 CNS1 F	CAGAGGTCAAAAGTGTGGGTATG
Foxp3 CNS1 R	ACTTGAGTTGAGGCTAGGTTGTTC
Foxp3 CNS2 F	CACATCCGCTAGCACCCACATCA
Foxp3 CNS2 R	TCATCGGCAACAAGGAGGAAGAGA
Foxp3 CNS3 F	GGGGCCCACACCTCTTCTTCCTT
Foxp3 CNS3 R	CCCCCGCCACATGCCACAGTAA
YY1 promoter F	TCAAAGAGCTAAATGCAAAGACTA
YY1 promoter R	ATTCCTCGCTCATAAACATTCC
CD25 promoter F	AGACAGCTTGGTGACACTATGAGAG
CD25 promoter R	GTGTTTACAGCTGCAATGGAAGA
Gitr promoter F	GGGGCAGAAGGGGGAAGG
Gitr promoter R	TGGCTAGGCTACAGAGGAAAAGGAT
Icos promoter F	CTATTTCAAAGGCCTTACATCTCTG
Icos promoter R	ATATCAATAGCTACTGGGGACTGC
Ctla4 promoter F	GGGGGATTAAAGATGACCAGATG
Ctla4 promoter R	AAGACGACCTCAAATATTCAAACTAAAA
β-globin F	AGATTTTTCCACTCCCTATTC
β-globin R	TGGCTGTAAGAAACCTAAATTAG

Supplementary Table 3. Primers used in qRT-PCR

Primer name	Sequences (5'→3')
Yy1 F	CAGTGGTTGAAGAGCAGATCAT
Yy1 R	AGGGAGTTTCTTGCCTGTCAT
Hprt F	FCTGGTGAAAAGGACCTCTCG
Hprt Probe	FAM-TGTTGGATACAGGCCAGACTTTGTTGGAT-TAMRA
Hprt R	TGAAGTACTCATTATAGTCAAGGGCA
Foxp3 F	GGCCCTTCTCCAGGACAGA
Foxp3 Probe	FAM-ACTTCATGCATCAGCTCTCCACTGTGGAT-TAMRA
Foxp3 R	GCTGATCATGGCTGGGTTGT
CD25 F	AACCATAGTACCCAGTTGTCGG
CD25 R	TCCTAAGCAACGCATATAGACCA
Ctla4 F	CAGGTGACCCAACCTTCAGT
Ctla4 R	CAGTCCTTGGATGGTGAGGT
Girt F	CGCGGGGAGCAGACAGAAGAA
Girt R	GGCCCAAAGACCCTACTCCAACAG
Icos F	TGACCCACCTCCTTTTCAAG
Icos R	TTAGGGTCATGCACACTGGA
Il10 F	GGTTGCCAAGCCTTATCGGA
Il10 R	ACCTGCTCCACTGCCTTGCT
